# Measuring Implicit Approach–Avoidance Tendencies towards Food Using a Mobile Phone outside the Lab

**DOI:** 10.3390/foods10071440

**Published:** 2021-06-22

**Authors:** Anne-Marie Brouwer, Jasper J. van Beers, Priya Sabu, Ivo V. Stuldreher, Hilmar G. Zech, Daisuke Kaneko

**Affiliations:** 1 Department of Human Performance, TNO, 3769 DE Soesterberg, The Netherlands; Jasper.V.Beers@outlook.com (J.J.v.B.); priyasabu1998@hotmail.com (P.S.); ivo.stuldreher@tno.nl (I.V.S.); 2 Department of Psychology and Neuroimaging Center, Technische Universität Dresden, 01069 Dresden, Germany; hilmar.zech@tu-dresden.de; 3 Kikkoman Europe R&D Laboratory B.V., 6709 PA Wageningen, The Netherlands; d.kaneko@kikkoman.nl

**Keywords:** food images, consumer, approach–avoidance, Approach–Avoidance Task (AAT), valence, arousal, wanting, implicit measure, self-report, mobile phone

## Abstract

Implicit (‘unconscious’) approach–avoidance tendencies towards stimuli can be measured using the Approach Avoidance Task (AAT). We recently expanded a toolbox for analyzing the raw data of a novel, mobile version of the AAT (mAAT), that asks participants to move their phone towards their face (pull) or away (push) in response to images presented on the phone. We here tested the mAAT reaction time and the mAAT distance in a study with 71 Dutch participants that were recruited online and performed an experiment without coming to the laboratory. The participants used both the mAAT and (explicit) rating scales to respond to photographic images of food. As hypothesized, the rated wanting, rated valence and mAAT reaction time indicated a preference for palatable over unpalatable food, and for Dutch over Asian food. Additionally, as expected, arousal was rated higher for unpalatable than for palatable food, and higher for Dutch than for Asian food. The mAAT distance indicated that the unpalatable food images were moved across larger distances, regardless of the movement direction (pull or push), compared to the palatable food images; and the Dutch food images were moved across larger distances than the Asian food images. We conclude that the mAAT can be used to implicitly probe approach–avoidance motivation for complex images in the food domain. The new measure of mAAT distance may be used as an implicit measure of arousal. The ratings and the mAAT measures do not reflect the exact same information and may complement each other. Implicit measures, such as mAAT variables, are particularly valuable when response biases that can occur when using explicit ratings are expected.

## 1. Introduction

Emotional attitudes towards food are considered to be important in predicting consumer behavior [1,2,3,4,5]. It has been shown that, compared to verbal liking preferences, food-evoked emotions have more predictive value in foreseeing whether consumers will like a product or not [1]. Recent literature reviews on the use of implicit (‘unconscious’) and explicit (self-report) methods to measure food-evoked emotions show the dominance of explicit methods in the field [6,7]. Implicit and explicit measures of food-evoked emotions can convey similar information. For instance, for a range of physiological, behavioral and explicit measures, responses toward tasting a clearly unpalatable drink stand out with respect to responses toward regular drinks [8]. However, on closer examination, all of these measures do reflect different processes. For instance, skin conductance has consistently been found to be positively associated with arousal [9,10,11,12], and is influenced by factors unrelated to emotion, such as temperature, whereas explicit reports on arousal reflect ‘arousal’ as interpreted by the individual, to the extent that he or she is aware of this and chooses to share this information. A difference between explicit and implicit measures, and thus, the added value of implicit measures, is, e.g., expected in cases of social pressure for a certain explicit response, or when explicit responses are affected by cultural bias [13,14,15].

The tendency to energize behavior towards a positive stimulus or away from a negative stimulus [16] is one of several facets of emotional experience. In the case of food, this approach–avoidance tendency can be estimated by asking individuals their explicit response to whether they want the food. As an implicit measure, Electroencephalogram (EEG) alpha asymmetry has been used [17,18]. Another implicit method, that does not rely on brain signals, is the Approach–Avoidance Task (AAT), first developed by Solarz [19]. He asked participants to pull cards towards themselves, or push them away, and found that cards with positive words were pulled more quickly than cards with negative words, and that cards with negative words were pushed more quickly than cards with positive words. When the original AAT was redesigned to run on personal computers [20,21], this greatly increased the flexibility of the task and facilitated its application across many different research areas. In the redesigned AAT, participants are presented with images on a computer screen and push these ‘away’ to avoid stimuli or pull them ‘near’ to approach stimuli by moving a joystick in the direction away or towards themselves, respectively. However, a downside of this version compared to the original, is the ambiguity introduced by the joystick. If one pulls a joystick to oneself, it is ambiguous whether that motion reflects the self (i.e., ‘moving myself away from the stimulus’, indicating avoidance) or whether the motion reflects the stimulus (i.e., ‘moving the stimulus to me’, indicating approach). Thus, for a more natural experience, reminiscent of the original test, yet easy to run and quantify, Zech et al. [22] developed a mobile version of the AAT (mAAT), in which images are presented on a smartphone screen that participants have to push away or pull toward themselves. Indeed, it was found that participants were faster when they had to approach positive stimuli (happy faces) or avoid negative stimuli (angry faces), compared to when these instructions were reversed [22]. The mAAT seems a particularly suitable tool to measure approach–avoidance in the domain of food, given that food has a very natural, unambiguous relation to approach and avoidance (bringing food to the mouth, or pushing it away). The fact that the mAAT runs on a mobile phone enables the collection of data outside the lab, which is useful for testing in specific contexts of interest [22] or when coming to the laboratory is impossible or inconvenient for other reasons, such as the COVID-19 pandemic.

As noted by Zech et al. [22], reaction time (RT) may not be the only variable of interest that can be extracted from the mAAT. Participants may not only respond quicker when moving a stimulus in the direction that is congruent to their (approach or avoidance) motivation but may also move these stimuli over a larger distance. The potential advantage of distance over RT is that it may be less sensitive to factors that can affect RT besides approach–avoidance motivation. In cases where complex stimuli are used, such a factor may be the time it takes to recognize a stimulus. We recently improved the usability and analysis of the data generated by the mAAT [23], including calculating the new variable of mAAT distance.

In the food domain, the AAT has been used to investigate healthy eating [24], food craving [25,26,27] and eating disorders [28]. There are few studies investigating the implicit AAT approach–avoidance tendencies related to food experience. A notable exception is [29]. In this study, a computerized joystick AAT paradigm was used on appealing and disgusting food images, wherein, as expected, the participants exhibited an approach bias towards appealing food and an avoidance bias away from disgusting food.

In the current study, we benchmarked the mAAT and the updated toolbox on photographic images of food. We utilized standardized images [30] for which a very strong difference in approach or avoidance motivation is expected: regular, palatable food (congruent with pull, incongruent with push), and food that was unpalatable because of mold or because it was infested by insects, worms or snails (congruent with push, incongruent with pull). We also used images for which a subtle difference in approach or avoidance motivation is expected: food from the participant’s own (in this case, Dutch) culture, and food from another culture (in this case, Asian). Previous studies consistently report that individuals overall prefer familiar food, or food from their own culture [13,15]. Both the mAAT RT and the mAAT distance were examined. The results were related to the explicit measures of approach–avoidance motivation (ratings of wanting) and emotion (valence and arousal) in response to the same set of images.

## 2. Materials and Methods

### 2.1. Participants

Participants were recruited through Prolific (www.prolific.co, Prolific, London, UK). In order to participate, participants had to have a Dutch nationality, fall within an age range of 18 to 65 years old and not follow any diet or suffer from any food allergy. See Supplementary File A for the recruitment text. A total of 120 individuals started the procedure. Complete datasets were obtained for 71 participants and were included in the analysis. Thirty of them were female, and their age ranged from 18 to 59, with a median of 30 years old. Their Body Mass Index ranged from 16.5 to 35.5, with a median of 24.5. Most of the participants reported eating Asian food weekly (*n* = 33), followed by monthly (*n* = 24). One participant reported eating Asian food every other day, and the remaining participants (*n* = 13) less than once a month. Participants who completed the experiment received a monetary reward of GBP 5. 

### 2.2. Materials

#### 2.2.1. Stimuli

**Figure 1:**
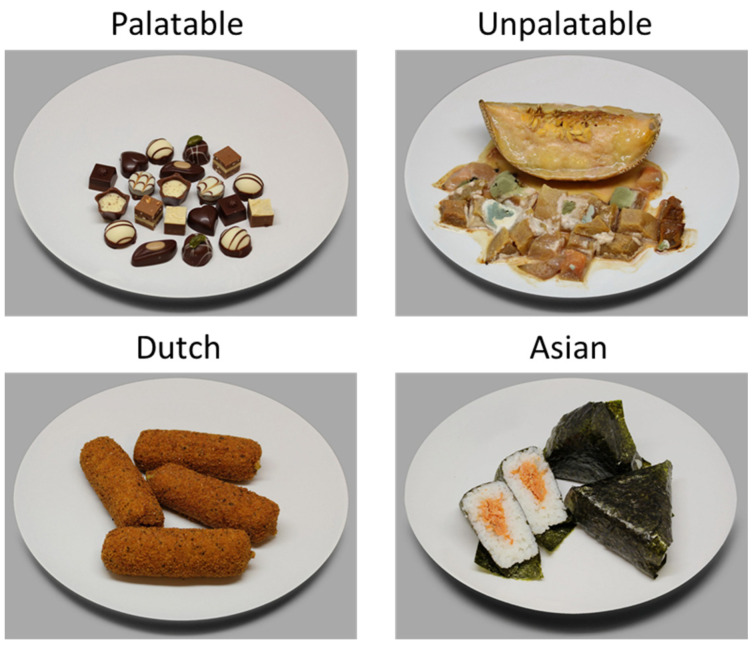
Example image from each of the four food image categories. Each category was represented by 20 unique images.

Food images were taken from the CROCUFID (CROss CUltural Food Images Database; [30]) and represented the following four categories: Asian food, Dutch food, palatable food (i.e., universal food, such as fruits and vegetables) and unpalatable food (i.e., molded food, or food with snails or insects crawling on it). Each category was represented by 20 unique images. Figure 1 shows an example image from each category. The complete set of used images is in Supplementary B.

#### 2.2.2. Questionnaires

Before the presentation of the food images, the participants filled out a questionnaire that was used to describe the participant sample and to enable the control of possibly relevant factors (such as current feelings of satiation and frequency of eating Asian food—see Supplementary File C). For the same reasons, they also filled out the Food Neophobia Scale [31], consisting of ten questions that the participant rated on a 7-point scale, ranging from ‘strongly disagree’ to ‘strongly agree’. High scoring participants are considered food neophobic, meaning that they are unwilling to try new food, while low scoring participants are enthusiastic about trying new and different food. We used Gorilla (www.Gorilla.sc, Cauldron Science, Cambridge, UK, accessed on 1 June 2020) as the experimental platform to ask the questions and direct the participants through the experiment.

#### 2.2.3. Stimulus Rating Scales

Each food image was rated using two rating scales. We used the EmojiGrid tool [32] to measure explicit food-related valence and arousal. The EmojiGrid is a 2D pictorial scale that separates the valence (*x*-axis) and arousal (*y*-axis) axes of emotion. To respond, participants click anywhere on the plane to express their food-related experience. For each trial, we recorded valence and arousal. Participants rated food wanting by using a slider on a VAS (Visual Analogue Scale) running from ‘fully disagree’ to ‘fully agree’ in response to the question ‘I want this very much’.

#### 2.2.4. mAAT

The mAAT app developed by Zech et al. [22] was set up for our conditions and made available for download from the Google Play Store. Participants installed the app on their personal phone. The app presents images and records the accelerations and rotational rates (if gyroscopic sensors are present) of the phone.

### 2.3. Experimental Design

Participants performed the experiment in two halves, interleaved with a break during which they were asked to watch a 6-minute movie (One group of participants (*n* = 38) was asked to watch a movie about the making of Lego bricks; the other group (*n* = 33) was asked to watch a movie about the making of soy sauce. We suspected that Asian food might be liked better after watching the movie about soy sauce compared to the movie unrelated to food. Since no such effect was observed in any of the variables, in this study, we grouped the data for all analyses.) The experiment halves were identical except for the exact images used, where we divided each of the four sets of 20 images (palatable, unpalatable, Dutch, Asian) into two sets of 10. Which set was presented before the break, and which after the break, was counterbalanced across the participants. Each half consisted of (firstly) the rating task and (secondly) the mAAT.

In the rating task, participants rated the images, presented in random order, using firstly, the EmojiGrid and, secondly, the wanting VAS. The mAAT task consisted of the following two parts: first, the Dutch and Asian food images were presented and, second, the palatable and unpalatable. Before the start of each part, participants were instructed to pull the phone towards them upon presentation of one (randomly determined) type of stimulus (e.g., ‘Dutch’) and push the phone away upon presentation of the other stimulus type (‘Asian’). When all of the images had been shown twice, the opposite instruction was given (i.e., in the example, to pull the phone when an ‘Asian’ food image was shown and push when ‘Dutch’ food was presented). Again, all of the images were shown twice. Thus, in the mAAT task, each of the images was presented four times; twice with the instruction to pull and twice with the instruction to push each image. Then, the part with the palatable and unpalatable food images was performed in the same way. After the break, the second half was performed.

### 2.4. Procedure

**Figure 2:**
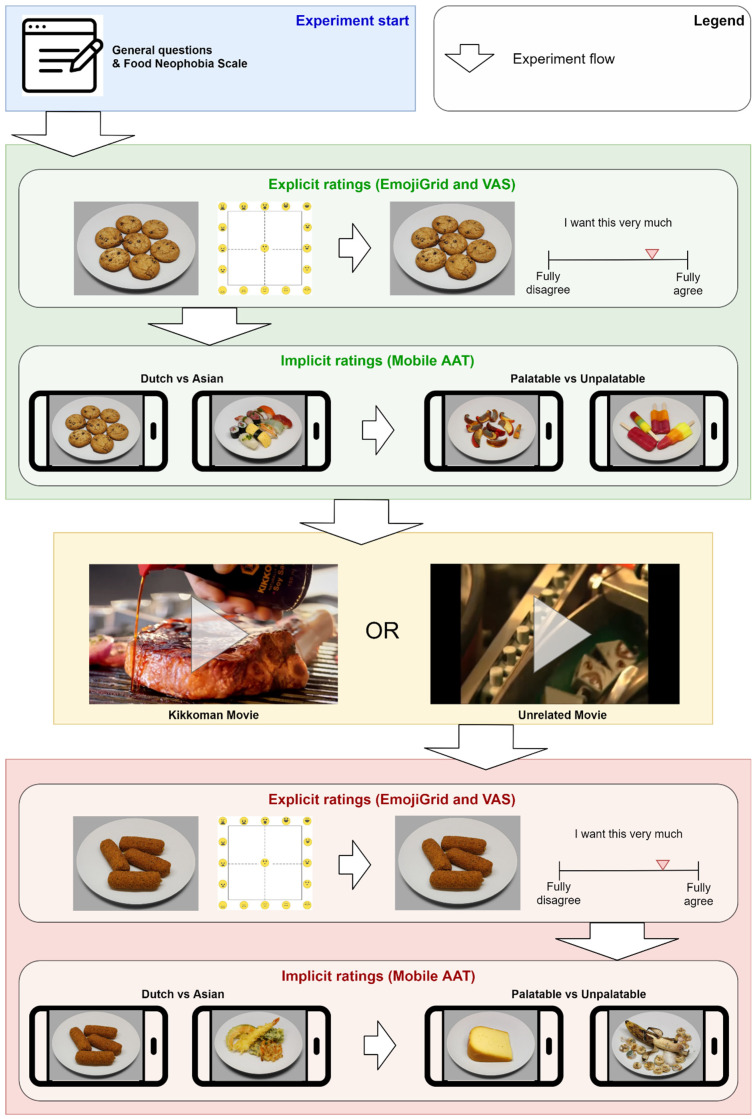
Schematic depiction of the experimental procedure.

Figure 2 depicts the procedure of the complete experiment. Participants read about the experiment in Prolific and signed the informed consent by clicking a checkbox. They could not proceed before giving informed consent. They were then instructed to download the mAAT app from the Appstore on their phone and were redirected to Gorilla on their (desktop or laptop) computer. Participants completed the general questionnaire and Food Neophobia Scale. After that, instructions appeared regarding the rating scales, asking participants to indicate their first impression. Then, the first half of the experiment started. Participants started with rating the food images using the explicit tools. Each image was first presented alongside the EmojiGrid. After clicking the location on the grid that best represented their current emotion towards the presented stimulus using the computer mouse or touchpad, the image was presented again alongside the wanting VAS. After clicking the appropriate location, the next image and scale appeared until all 40 images were rated. Participants were then instructed on the mAAT, including a short movie of the desired type of movements. For each of the four combinations of food types (Asian/Dutch, palatable/unpalatable) and movement instruction (pull ‘A’/push ‘B’ or pull ‘B’/push ‘A’), participants practiced 5 trials with a dedicated set of (CROCUFID) images from the relevant food categories that were distinct from those used in the experimental trials. Within each trial, participants first saw a fixation cross for 500 ms to guide the eyes to the center of the phone’s display. After this, the current trial’s image was shown until either the participant responded by moving the phone, or after 2 s had elapsed (this was considered as ‘no reaction’). After pushing or pulling the phone, participants completed the response by immediately returning the phone to the initial position. Once the phone had come to rest, the next trial started. After finishing the mAAT, participants were directed to their computer to watch a movie as a break. Then, the second half of the experiment started, which was identical to the first, except for the exact images used. The whole procedure took about 1 h to complete.

### 2.5. Analysis

For each participant and each stimulus category (palatable, unpalatable, Dutch, Asian), an average score of EmojiGrid valence, EmojiGrid arousal and rated wanting was determined. Wilcoxon signed ranks tests were used to test for significant differences between the palatable and unpalatable, and between the Asian and Dutch food images.

Data from the mAAT app were processed using the expanded mAAT processing toolbox, as described in [23]. The toolbox is freely available for download at https://github.com/Jasper-van-beers/AAT (accessed on 30 November 2020). The mAAT RTs were defined as the time between stimulus onset and onset of the motion of the phone. Motion onset was defined as the moment that the acceleration is greater than the maximum (0.8, (0.3∙a_max_)) ms^−2^, with a_max_ denoting the maximum measured acceleration. Any RTs < 200 ms were discarded and any RTs > 2000 ms were considered to be ‘no reactions’. Data from participants with less than 75% valid trials were considered to be incomplete datasets and were not included in the analyses. The innovative feature of mAAT distance was derived using the magnitude and the duration of the acceleration.

An average RT and an average distance were calculated for each participant, stimulus category and movement direction (pull or push). Repeated measure ANOVAs with stimulus category and movement direction were applied to the mAAT RT and the mAAT distance for the palatable and unpalatable food images, and for the Asian and Dutch food images.

To further explore how implicit mAAT responses relate to other measures that we expect to be associated with the approach and avoidance motivation, we computed an mAAT RT score by subtracting ‘mAAT RT pull’ from ‘mAAT RT push’ for each participant and each image category. A high mAAT score would correspond to approach motivation. It was expected to correlate positively with valence and wanting scores, and negatively with food neophobia for Asian food images. Pearson correlations were performed to test for these effects.

Repeated measure ANOVAs were performed using an SPSS 25 (IBM, Armonk, NY, USA). Wilcoxon signed ranks tests and Pearson correlations were performed using a MATLAB R2020a (The MathWorks Inc., Natick, MA, USA). For all statistical tests, we used an alpha level of 0.05.

## 3. Results

### 3.1. Explicit Ratings

**Figure 3:**
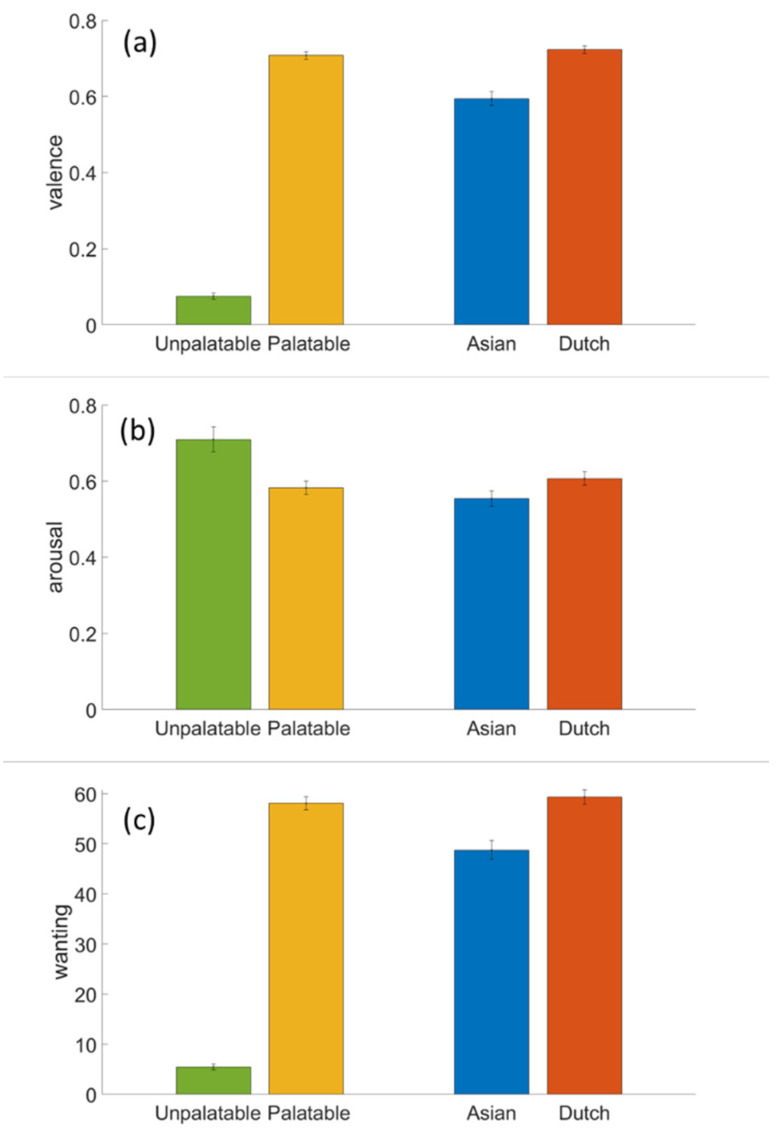
Explicit ratings valence (**a**), arousal (**b**) and wanting (**c**) for each of the four stimulus categories. Error bars indicate standard errors of the mean.

Figure 3 shows the explicit ratings of valence (a), arousal (b) and wanting (c), averaged across the participants for each of the four stimulus categories. The Wilcoxon signed ranks tests indicated significant differences between the palatable and unpalatable food images, and between the Asian and Dutch food images, for all three explicit ratings (all *p*-values < 0.01). The valence and wanting indicated a preference for palatable over unpalatable, and a preference for Dutch over Asian food. The rated arousal was higher for unpalatable than for palatable food, and higher for Dutch than for Asian food.

### 3.2. mAAT Measures

**Figure 4:**
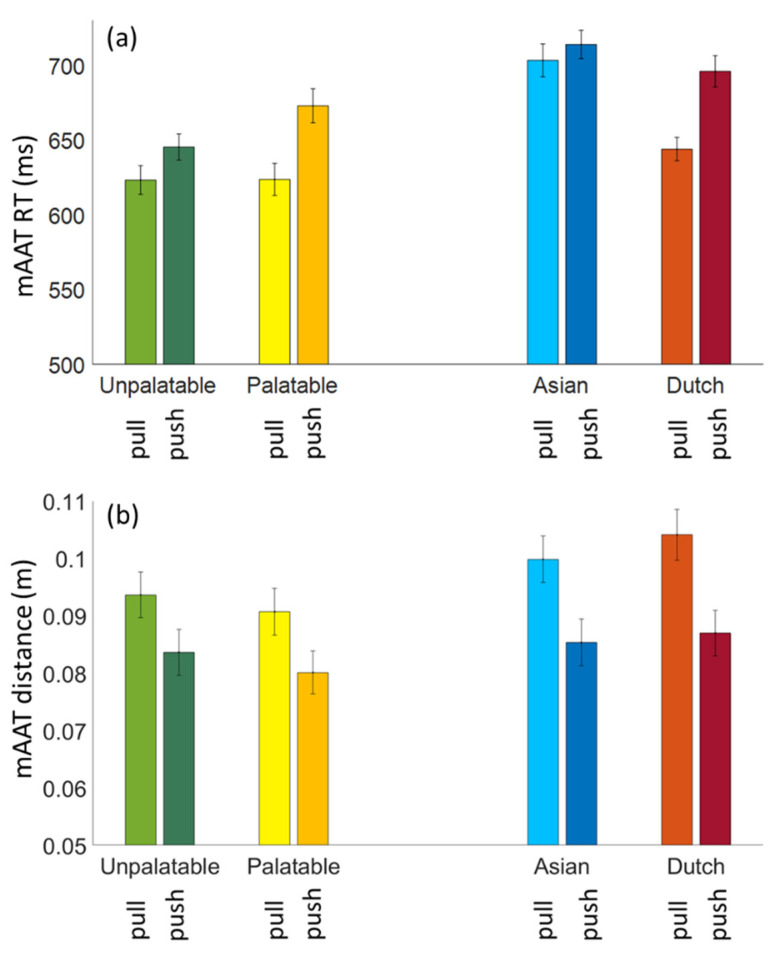
mAAT RT (**a**) and mAAT distance (**b**) for each of the four stimulus categories and each movement direction, pull and push.

Figure 4 shows the mAAT RT (a) and the mAAT distance (b) averaged across the participants for each of the four stimulus categories and the push–pull direction.

For the mAAT RT, the ANOVA for palatable and unpalatable food showed that, in general, people responded quicker when making a pulling than a pushing movement (main effect of movement direction: *p* < 0.001) and that responses to unpalatable food were quicker (main effect of image type: *p* < 0.001). Importantly, a significant interaction effect between the movement direction and the image type (*p* < 0.001) showed that, as expected, the participants were quicker to push a stimulus congruent with avoidance motivation (i.e., unpalatable food) than a stimulus that was not, relative to pulling. The explicit ratings and the literature led to the expectation that familiar food (Dutch) and unfamiliar food (Asian) result in similar mAAT tendencies as palatable and unpalatable food, respectively. Indeed, the ANOVA for the Asian and Dutch food images showed similar results, with a main effect of the movement direction (*p* < 0.001) and of the image type (*p* < 0.001), as well as an interaction effect (*p* = 0.007), indicating quicker pulling responses than pushing, but especially for the Dutch food images.

For the mAAT distance, the ANOVA for palatable and unpalatable food showed a significant main effect of the movement direction (*p* < 0.001), with shorter distances for pushing than pulling, and a significant effect of the image type (*p* < 0.001), indicating that, overall, the unpalatable food images were moved across larger distances than the palatable images. There was no interaction (*p* = 0.79). The same pattern of results was found for Dutch and Asian food, with a main effect of the movement direction (*p* < 0.001), and a main effect of the image type (*p* = 0.001), where the Asian food images were moved across larger distances compared to the Dutch food images. No interaction effect was present (*p* = 0.99).

### 3.3. Correlations

**Figure 5:**
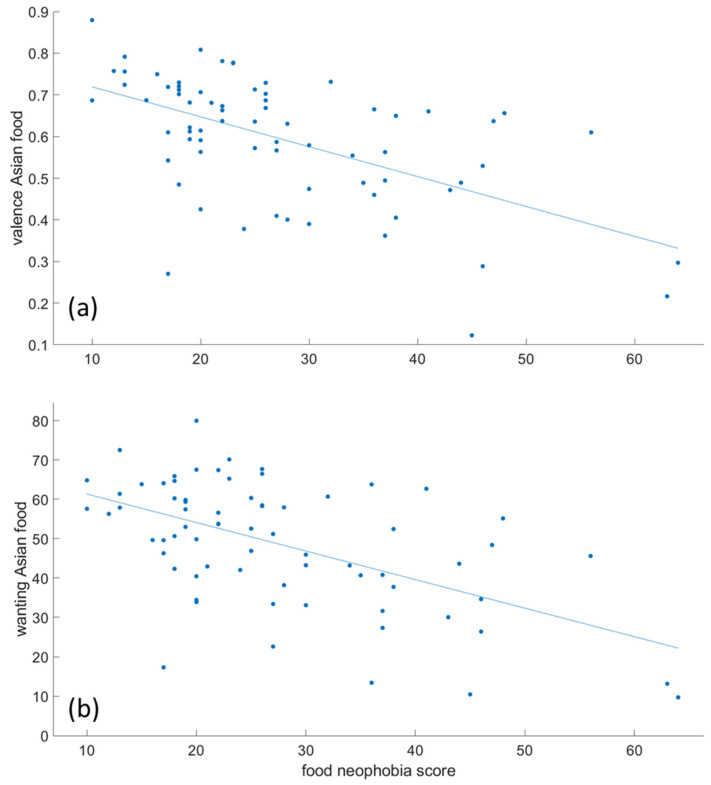
Correlation between food neophobia score and EmojiGrid valence (**a**) and wanting (**b**) for Asian food images. Each data point represents one participant.

The mAAT RT score did not significantly correlate with valence or wanting for any of the four food image categories. It also did not correlate with food neophobia for Asian food images. As a comparison, food neophobia did show a negative correlation with the EmojiGrid valence for Asian food images (*R*^2^ = 0.32, *p* < 0.001; Figure 5a), and a similar negative correlation was found between food neophobia and wanting (*R*^2^ = 0.31, *p* < 0.001; Figure 5b).

Given the effects of the food image categories on the mAAT distance and arousal, we computed an mAAT distance score by averaging the pull and push distance per image category and per participant. These values were correlated to rated arousal for each image category separately, but no significant relations were found.

## 4. Discussion

The current study showed that approach–avoidance tendencies for food can be reliably measured in participants in the field using a phone, without personal technical help or instructions.

The mAAT RT results showed the expected interaction between an image category and a movement direction, not only for the stimulus categories that were expected to differ strongly in approach–avoidance motivation (palatable and unpalatable food images), but also for more subtly differing food categories (images depicting food from the participant’s own or another culture). The explicit ratings of valence, arousal and wanting showed the expected pattern of a strong preference for palatable over unpalatable food, and a preference for their own culture’s (Dutch) food over another culture’s (Asian) food. While our design did not allow for a direct statistical comparison, as one would expect, the size of the effect in the mAAT RT (i.e., the difference between pull and push), seems to be similar for the palatable and the Dutch food images, whereas the effect seems to be larger for the unpalatable than for the Asian food images. The overall shorter RTs to the palatable and the unpalatable food images compared to the Dutch and the Asian food images may be explained by the difference in the time it takes to identify and categorize the images. It may also be a time order (practice) effect—in each of the two experiment halves, participants responded to the Dutch and Asian food images before the palatable and unpalatable images.

The mAAT distance results showed a different pattern than the mAAT RT results. We had anticipated the mAAT distance to mirror the mAAT RT, i.e., the food images congruent with approach may be pulled both quicker and further towards oneself, and the images congruent with avoidance would be pushed both quicker and further away, where distance may have been relatively unaffected by aspects that are expected to affect RT, such as recognition of the stimulus. However, what we found were larger distances for the unpalatable and the Dutch food images, irrespective of the movement’s direction. The unpalatable and the Dutch food images were also judged relatively high in arousal (as found before [15]). Given the specific food images used, depicting molded and infested food, high arousal for the unpalatable images does not come as a surprise. The finding that the Dutch food images were rated higher in arousal than the Asian ones can be understood by the fact that both types of images were generally rated as pleasant, in which case valence and arousal are commonly found to be positively related [33,34,35]. Since Dutch food is rated high in valence, the high arousal scores are not surprising. The finding that the mAAT distance may be associated with arousal is intriguing and important, since it has been argued that arousal is a crucial determinant in determining (sustained) the attractiveness of products [36,37], but is also hard to capture with explicit questionnaires [35,38]. It would also nicely complement the mAAT RT approach–avoidance motivation, that is more closely related to valence. Future studies need to replicate and further test the possible association between the mAAT distance and arousal.

Given the previous and current results, correlations between rated wanting and mAAT RT, as well as between rated arousal and mAAT distance, may have been expected. However, we did not find such correlations at the participant and stimulus category level. This suggests that these (explicit and implicit) measures reflect different processes. A discrepancy at the condition level may be observed if a discrepancy between the explicit and implicit measures is expected, such as may be the case when there is social pressure to shape explicit responses in a certain way.

A limitation of the study is the loss of participants and data. Twenty-four of the 120 participants that started the procedure quit after performing only a fraction of the experiment. Some of them may not have been able to generate proper mAAT movements. Another twelve participants did not reach the criterion of 75% valid mAAT trials. The number of valid mAAT trials may be increased in the future by setting more strict inclusion criteria for the phones that can be used (e.g., only those containing a linear accelerometer) and by giving participants more precise feedback about inappropriate movements (e.g., rotations rather than pulling and pushing) during the test. In our study, the data of another 13 participants were lost because they did not fill out the rating scales and questionnaires completely or filled out information incompatible with the inclusion criteria. Future online experiments can be made more robust against such omissions by preventing participants from proceeding whenever data is missing or incompatible.

## 5. Conclusions

In conclusion, the current study showed the sensitivity of the mAAT to measure an approach–avoidance motivation to complex food images, and with the new measure of mAAT distance, possibly arousal, therewith complementing the dominant use of explicit tools in research on food experience. The mAAT more closely maps onto approach–avoidance movement than joystick approaches do. Moreover, the mAAT is a promising tool for evaluating food experience, since it can be used to collect users’ implicit tendencies remotely, which can be valuable both from a practical point of view and from a research perspective, when research questions are related to specific times and places that are not compatible with laboratory tests.

## Supplementary Materials

The following are available online at https://www.mdpi.com/article/10.3390/foods10071440/s1, Supplementary A: Recruitment, Supplementary B: FoodImages, Supplementary C: Questionnaire.
