# Supplementary material for: Measuring Implicit Approach–Avoidance Tendencies towards Food Using a Mobile Phone outside the Lab
Source: Foods. 2021 Jun 22;10(7):1440. doi: 10.3390/foods10071440 (PMC8305314; doi:10.3390/foods10071440)
Supplement: Supplementary file 1 [file foods-10-01440-s001.zip › SupplementaryFiles/SuppARecruitment.pdf]

A study on Food Experience

In this experiment you will see 80 food images and a short video. We ask you to rate these images through two different response tools. Part of this experiment involves responding through a mobile app, which we will provide a link to in the experiment.

Attention:

- You need **both** an **Android smartphone and a computer** (laptop or desktop) to participate in this experiment
- **Make a note of (or copy) your prolific ID** as you will need it for the experiment
- Please open this experiment in a new tab

About the experiment

The goal of the experiment is to explore and compare different types of responses to food images, e.g. responses that involve language and responses that do not. Results can be useful for studying food appreciation across cultures.

In this experiment you will see 80 food images and a short video.

We ask you to rate these images through two different response tools.

Part of this experiment involves responding to images through a mobile app, which we will provide a link to shortly.

Attention: The mobile app only works on Android devices, so you need an **Android smartphone** to participate in this experiment.

The experiment is structured as follows:

1. Questions regarding your demographics (including body length and weight) and food preferences (~10 minutes)
2. Image rating task on 40 images (~12 minutes)
3. Mobile app responses on 40 images (~10 minutes)
4. Short video (~6 minutes)
5. Image rating task on 40 (different from 2.) images (~12 minutes)
6. Mobile app responses on 40 (different from 3.) images (~10 minutes)

Requirements for taking part in this study:

- Age between 18-65 years
- Dutch nationality
- No food-allergies or specific diets

You are free to withdraw and stop at any point of the experiment. However please be aware that no reimbursement (6,60 euro) will be given if the experiment is not completed. Your responses will be collected anonymously and are kept confidential. This study involves no known or anticipated risks.
