# Supplementary material for: Measuring Implicit Approach–Avoidance Tendencies towards Food Using a Mobile Phone outside the Lab
Source: Foods. 2021 Jun 22;10(7):1440. doi: 10.3390/foods10071440 (PMC8305314; doi:10.3390/foods10071440)
Supplement: Supplementary file 1 [file foods-10-01440-s001.zip › SupplementaryFiles/SuppCQuestionnaire.pdf]

Questionnaire

General questions:

- What is your age (years)?
- What is your height (cm)?
- What is your weight (kg)?
- What is your nationality?
- Do you have food allergies?
    - If yes, please specify *[Free answer]*
- Are you on a diet (incl. vegan, vegetarian, pescatarian etc.) *[Free answer]*
- How often do you eat Asian food?
    - Daily, Every other day, weekly, Monthly, Less than once a month *[One option]*
- How hungry are you right now?
    - Not hungry at all - Very hungry *[VAS]*
- How thirsty are you right now?
    - Not thirsty at all - Very thirsty *[VAS]*
- How full do you feel right now?
    - Not full at all - Very full *[VAS]*
- How much time did pass since your last food consumption (hours)? *[Free answer]*

FNS

[Likert scale with: Strongly Disagree, Disagree, Somewhat Disagree, Neutral, Somewhat Agree, Agree, Strongly Agree]

- I am constantly sampling new and different foods.
- I don’t trust new foods.
- If I don’t know what is in a food, I won’t try it.
- I like foods from different countries.
- Ethnic food looks too weird to eat.
- At dinner parties, I will try new food.
- I am afraid to eat things that I have never had before.
- I am very particular about the foods I will eat.
- I will eat almost anything.
- I like to try new ethnic restaurants.
