# Supplementary figures and images for: Measuring Implicit Approach–Avoidance Tendencies towards Food Using a Mobile Phone outside the Lab
Source: Foods. 2021 Jun 22;10(7):1440. doi: 10.3390/foods10071440 (PMC8305314; doi:10.3390/foods10071440)

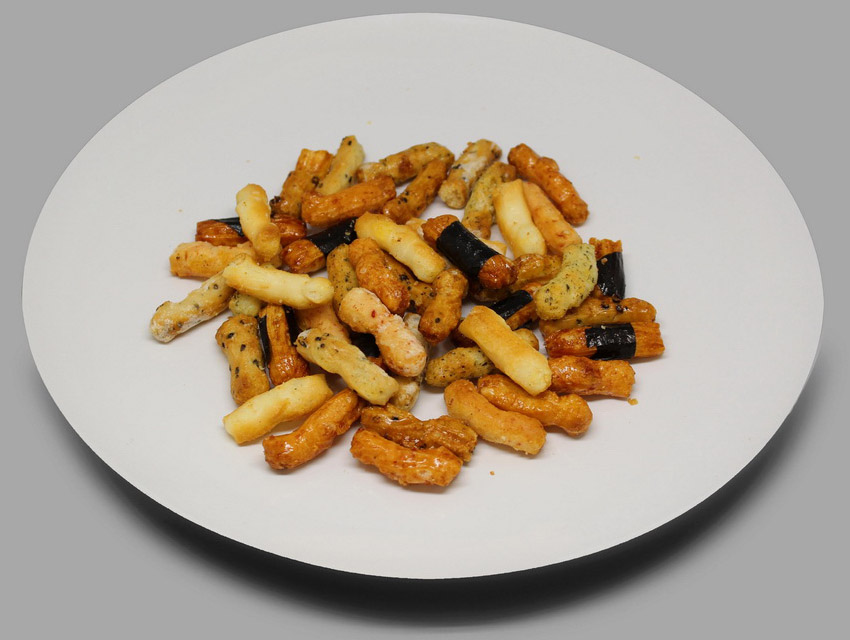

Supplement: Supplementary file 1 [file foods-10-01440-s001.zip › SupplementaryFiles/SuppBFoodImages/SubsetA/Asian/tp0359.jpg]

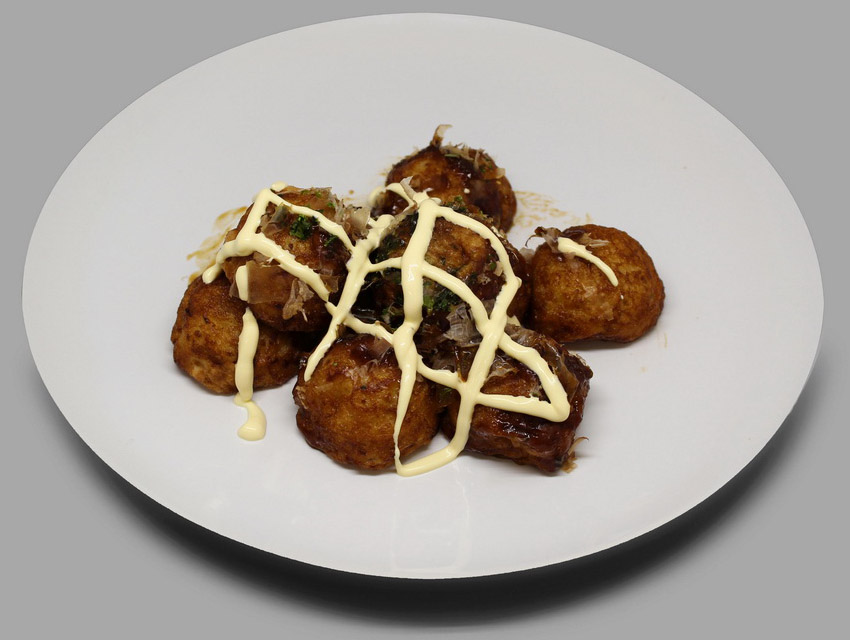

Supplement: Supplementary file 1 [file foods-10-01440-s001.zip › SupplementaryFiles/SuppBFoodImages/SubsetA/Asian/tp0379.jpg]

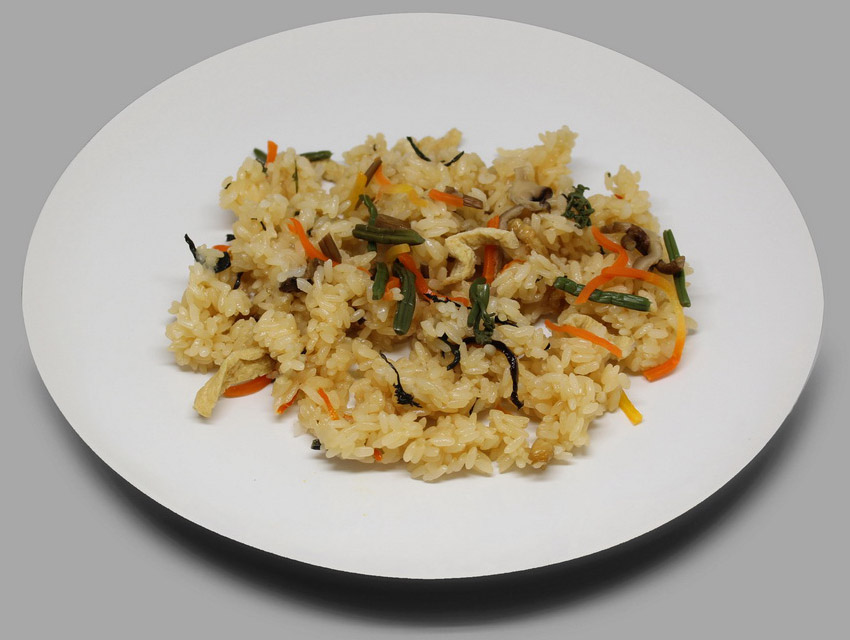

Supplement: Supplementary file 1 [file foods-10-01440-s001.zip › SupplementaryFiles/SuppBFoodImages/SubsetA/Asian/tp0397.jpg]

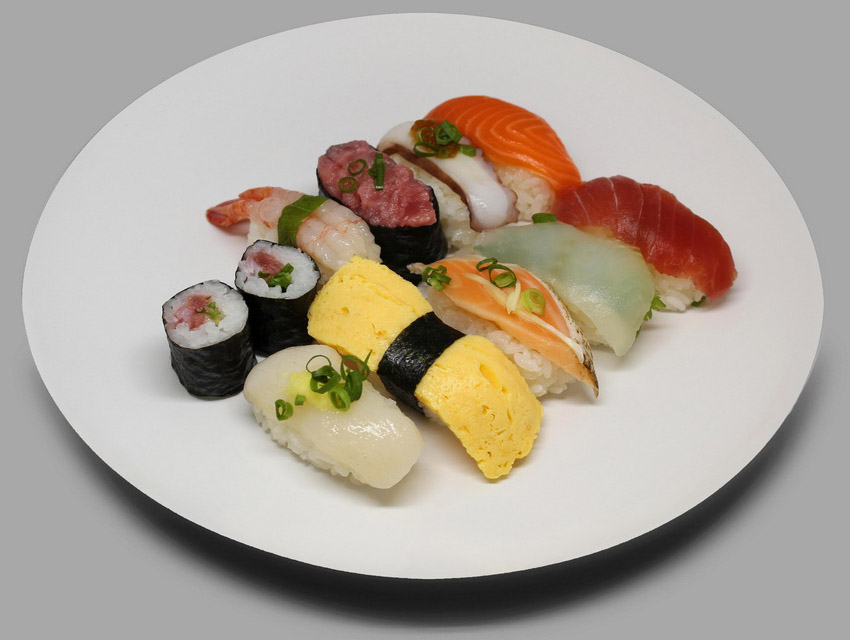

Supplement: Supplementary file 1 [file foods-10-01440-s001.zip › SupplementaryFiles/SuppBFoodImages/SubsetA/Asian/tp0412.jpg]

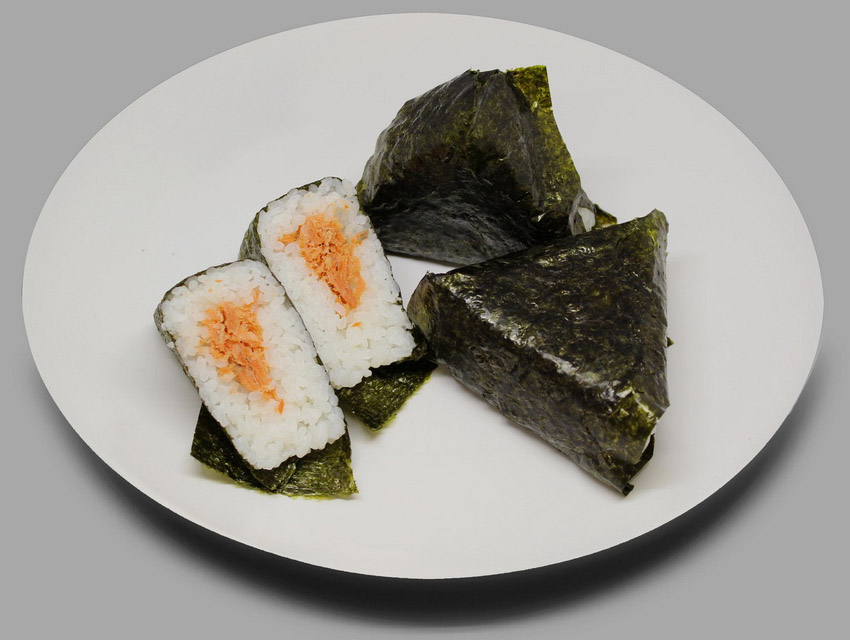

Supplement: Supplementary file 1 [file foods-10-01440-s001.zip › SupplementaryFiles/SuppBFoodImages/SubsetA/Asian/tp0427.jpg]

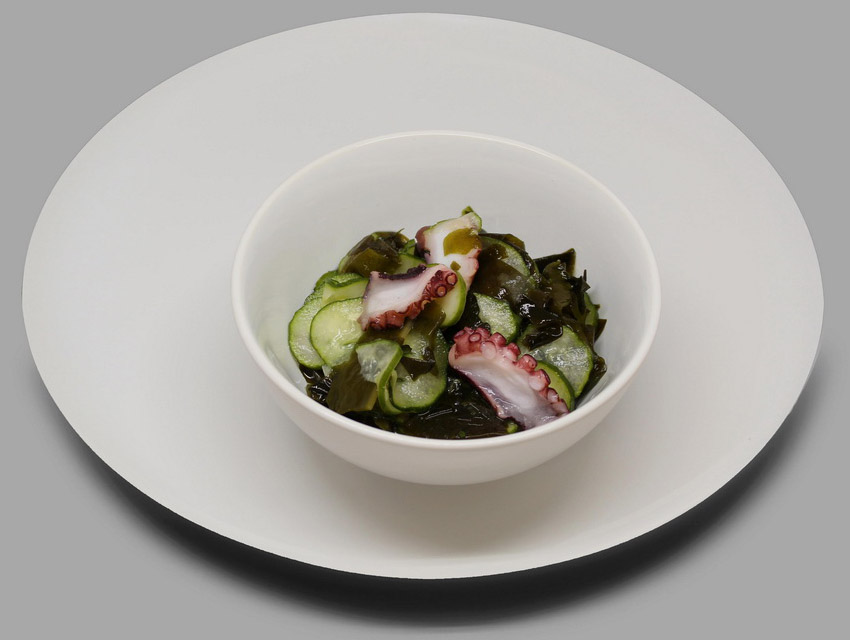

Supplement: Supplementary file 1 [file foods-10-01440-s001.zip › SupplementaryFiles/SuppBFoodImages/SubsetA/Asian/tp0435.jpg]

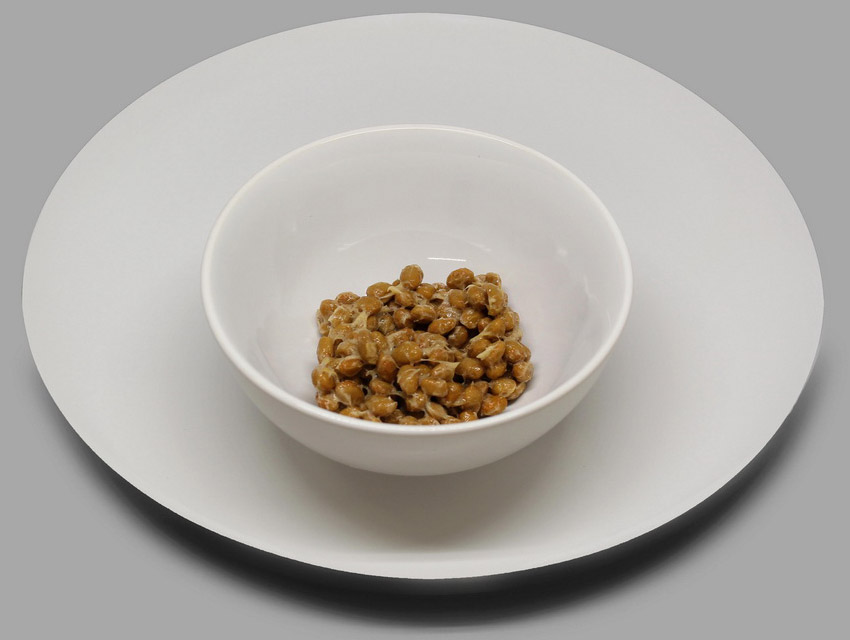

Supplement: Supplementary file 1 [file foods-10-01440-s001.zip › SupplementaryFiles/SuppBFoodImages/SubsetA/Asian/tp0479.jpg]

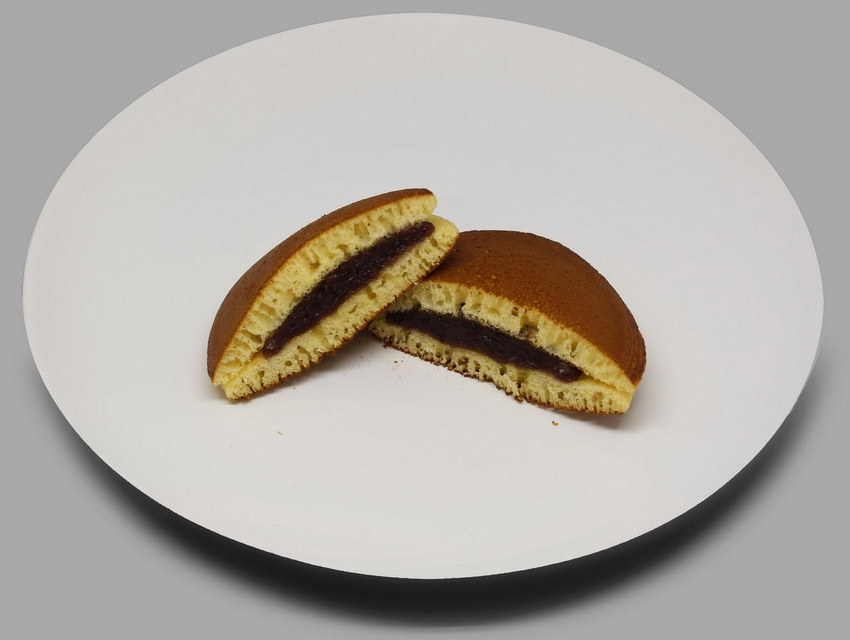

Supplement: Supplementary file 1 [file foods-10-01440-s001.zip › SupplementaryFiles/SuppBFoodImages/SubsetA/Asian/tp0496.jpg]

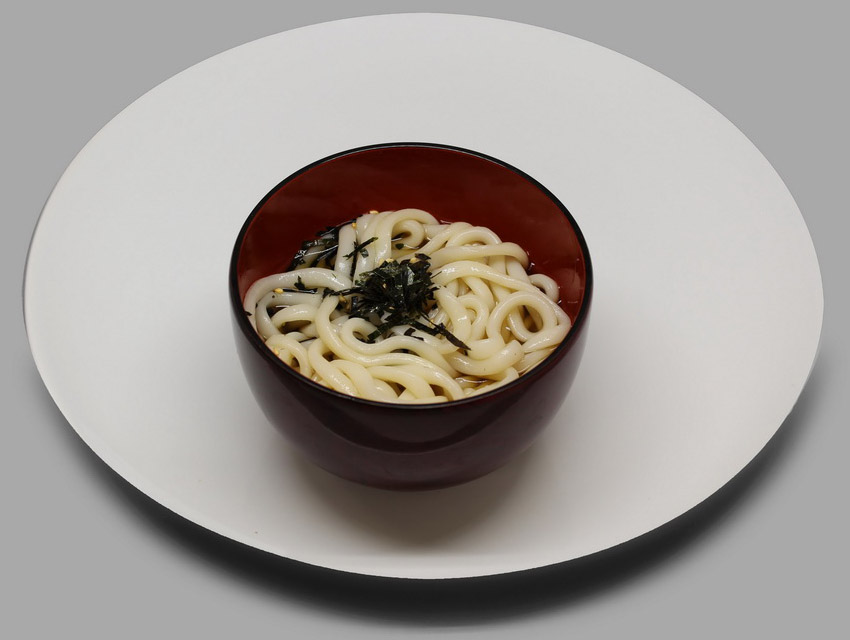

Supplement: Supplementary file 1 [file foods-10-01440-s001.zip › SupplementaryFiles/SuppBFoodImages/SubsetA/Asian/tp0503.jpg]

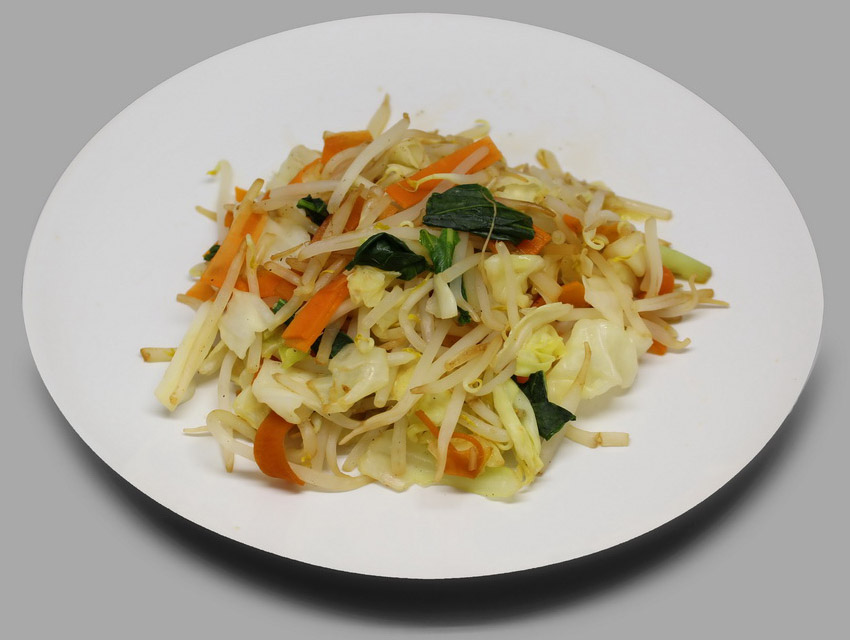

Supplement: Supplementary file 1 [file foods-10-01440-s001.zip › SupplementaryFiles/SuppBFoodImages/SubsetA/Asian/tp0524.jpg]

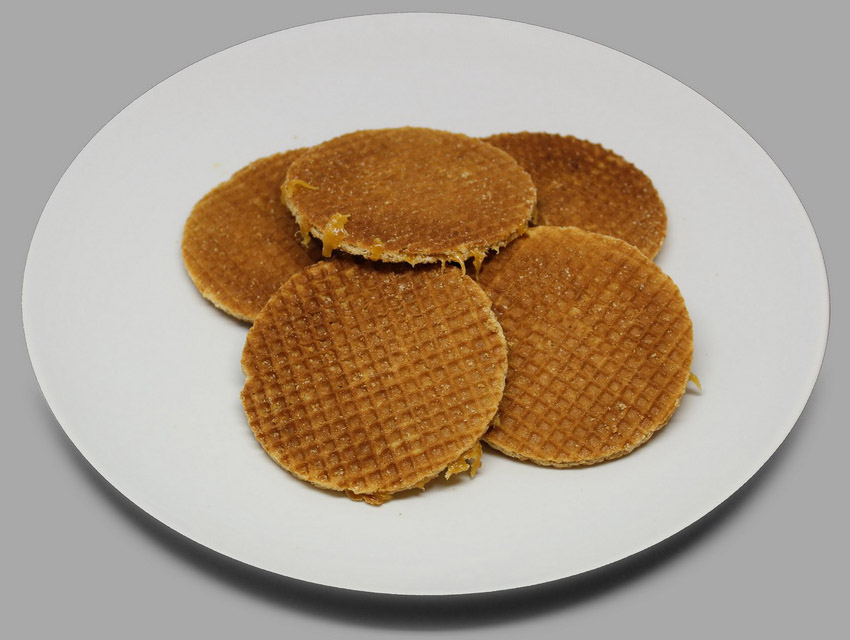

Supplement: Supplementary file 1 [file foods-10-01440-s001.zip › SupplementaryFiles/SuppBFoodImages/SubsetA/Dutch/tp0021.jpg]

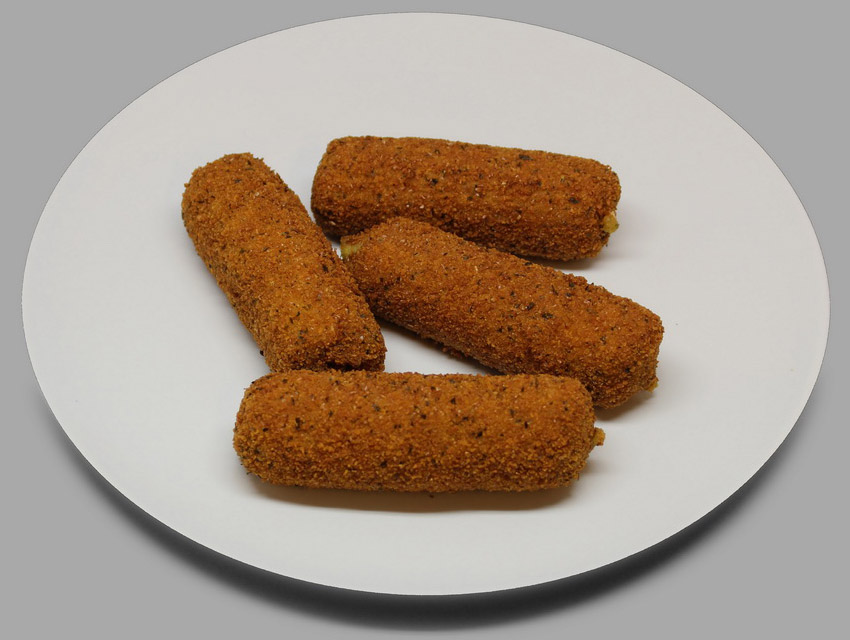

Supplement: Supplementary file 1 [file foods-10-01440-s001.zip › SupplementaryFiles/SuppBFoodImages/SubsetA/Dutch/tp0248.jpg]

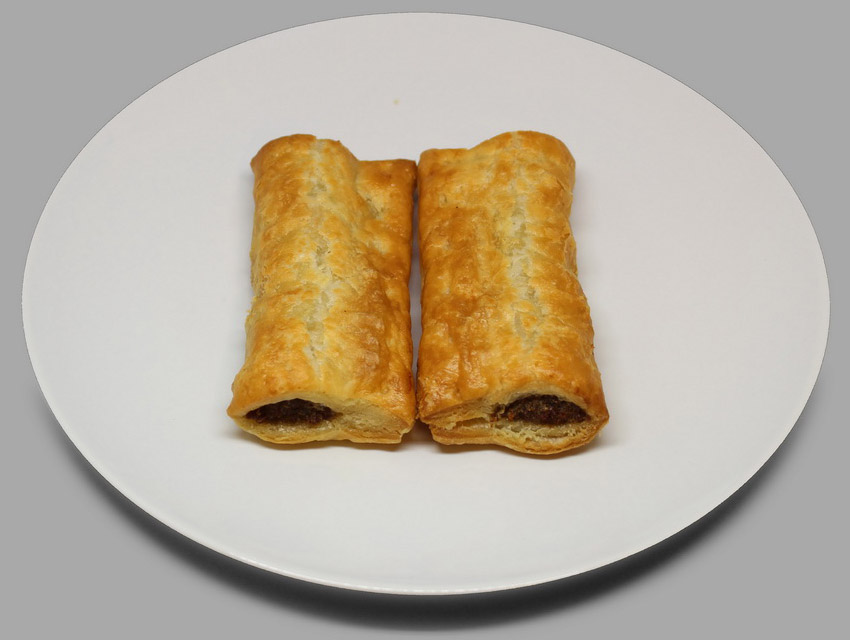

Supplement: Supplementary file 1 [file foods-10-01440-s001.zip › SupplementaryFiles/SuppBFoodImages/SubsetA/Dutch/tp0335.jpg]

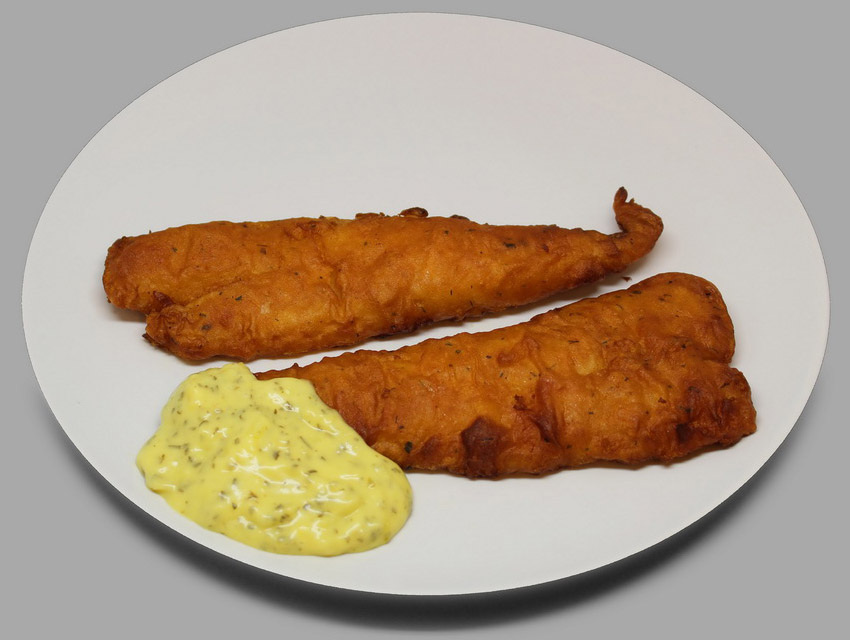

Supplement: Supplementary file 1 [file foods-10-01440-s001.zip › SupplementaryFiles/SuppBFoodImages/SubsetA/Dutch/tp0338.jpg]

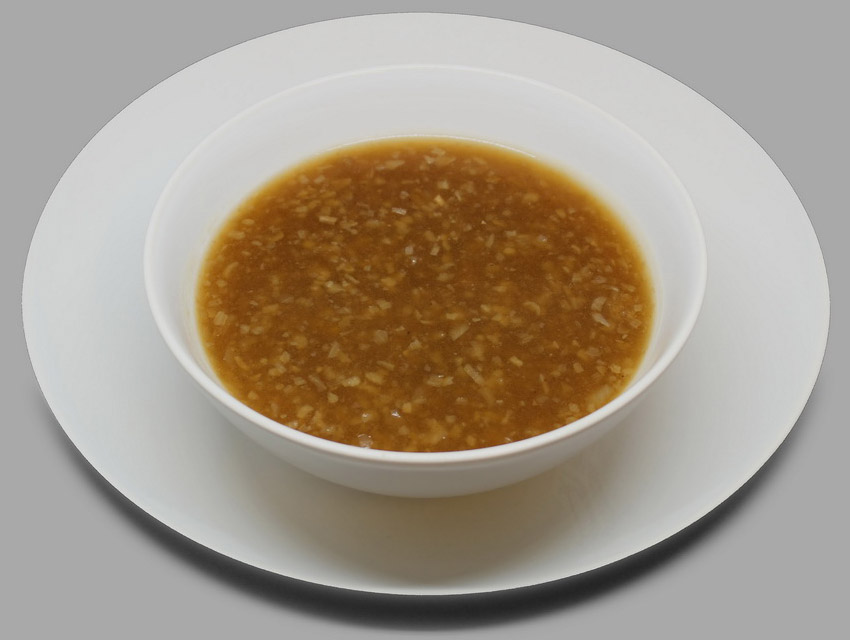

Supplement: Supplementary file 1 [file foods-10-01440-s001.zip › SupplementaryFiles/SuppBFoodImages/SubsetA/Dutch/tp0344.jpg]

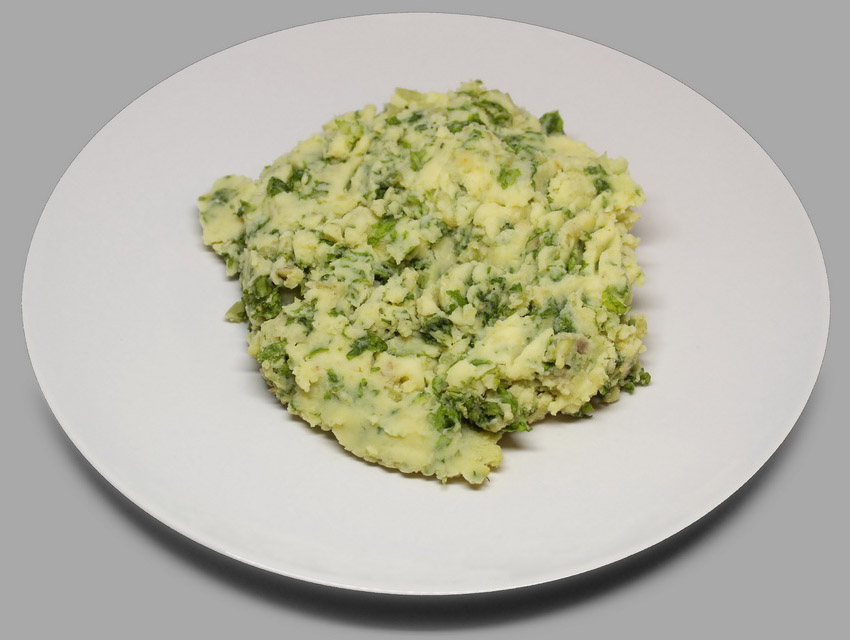

Supplement: Supplementary file 1 [file foods-10-01440-s001.zip › SupplementaryFiles/SuppBFoodImages/SubsetA/Dutch/tp0349.jpg]

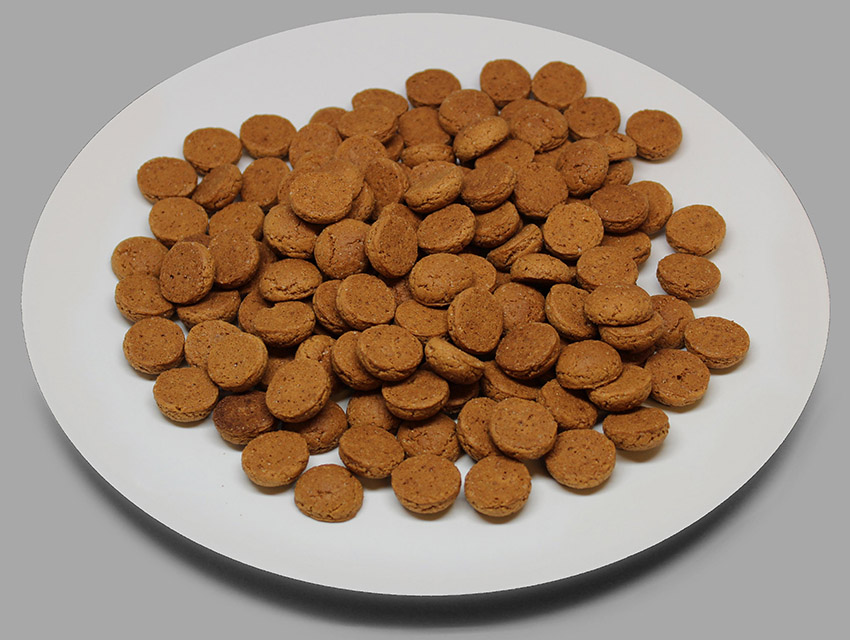

Supplement: Supplementary file 1 [file foods-10-01440-s001.zip › SupplementaryFiles/SuppBFoodImages/SubsetA/Dutch/tp0837.jpg]

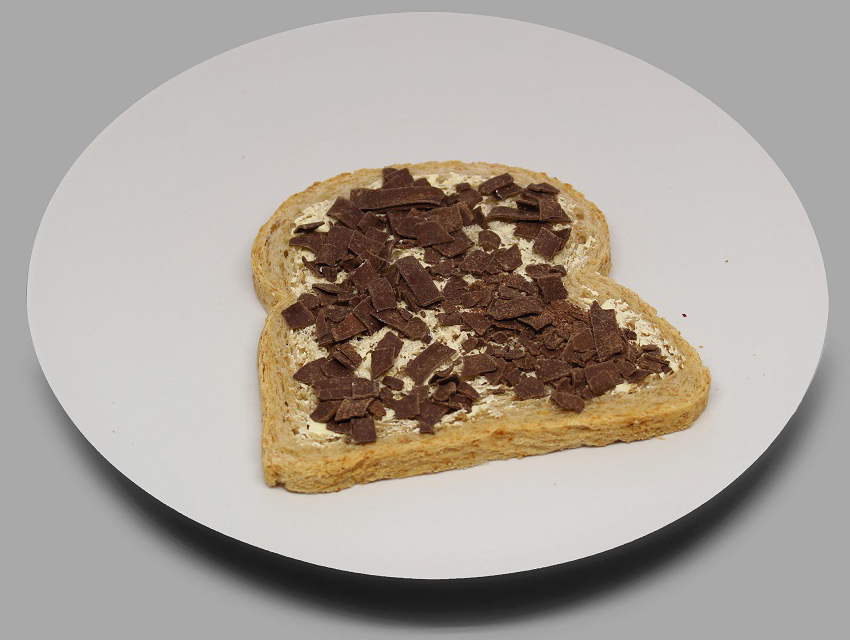

Supplement: Supplementary file 1 [file foods-10-01440-s001.zip › SupplementaryFiles/SuppBFoodImages/SubsetA/Dutch/tp0857.jpg]

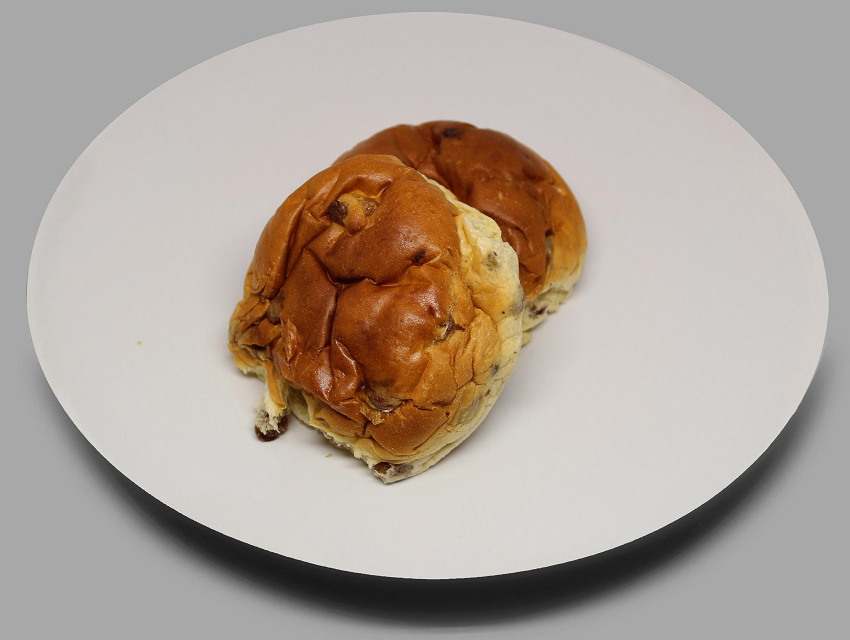

Supplement: Supplementary file 1 [file foods-10-01440-s001.zip › SupplementaryFiles/SuppBFoodImages/SubsetA/Dutch/tp0870.jpg]

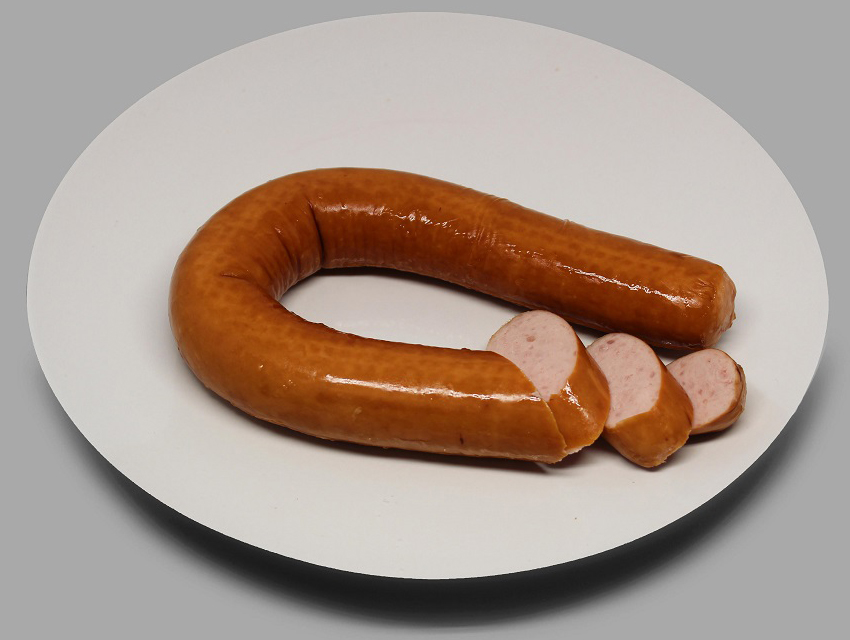

Supplement: Supplementary file 1 [file foods-10-01440-s001.zip › SupplementaryFiles/SuppBFoodImages/SubsetA/Dutch/tp0880.jpg]

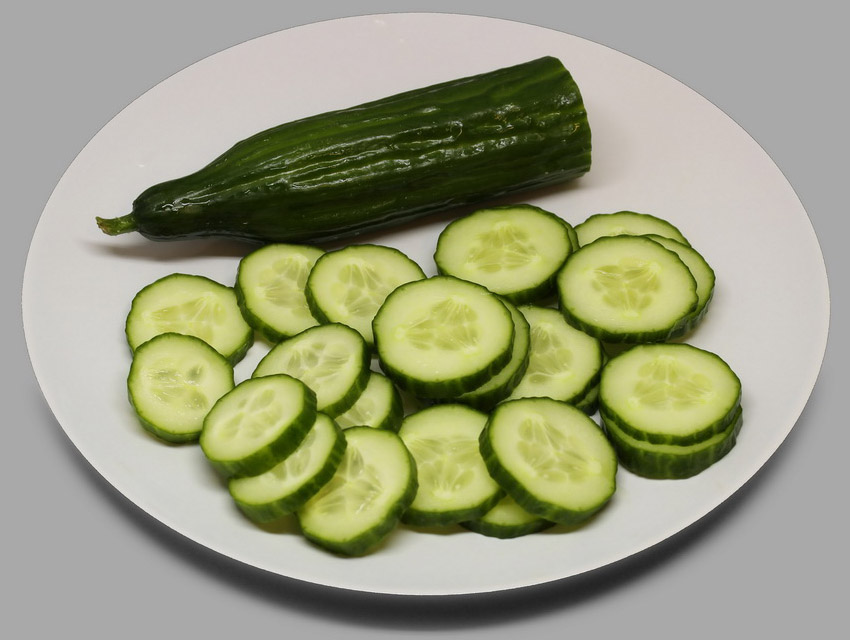

Supplement: Supplementary file 1 [file foods-10-01440-s001.zip › SupplementaryFiles/SuppBFoodImages/SubsetA/Palatable/tp0036.jpg]

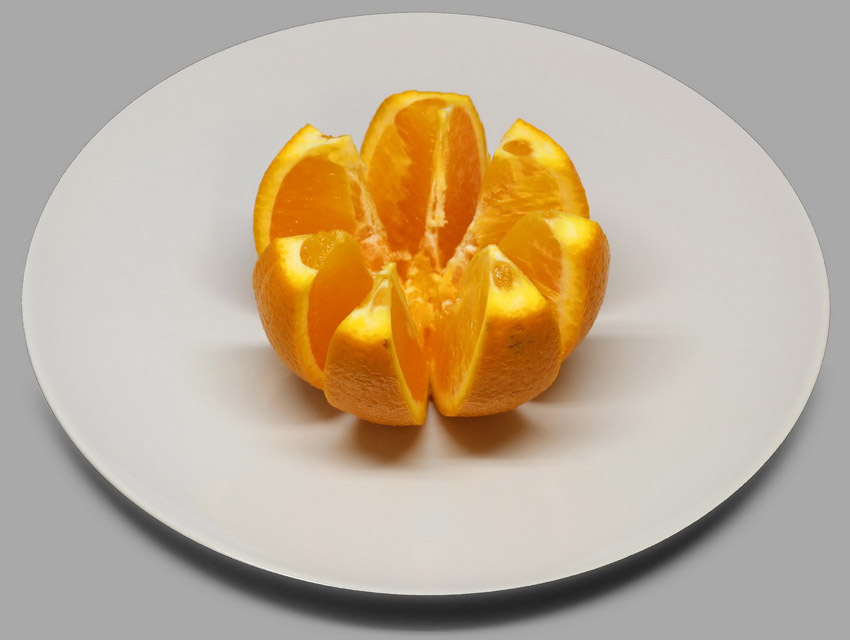

Supplement: Supplementary file 1 [file foods-10-01440-s001.zip › SupplementaryFiles/SuppBFoodImages/SubsetA/Palatable/tp0043.jpg]

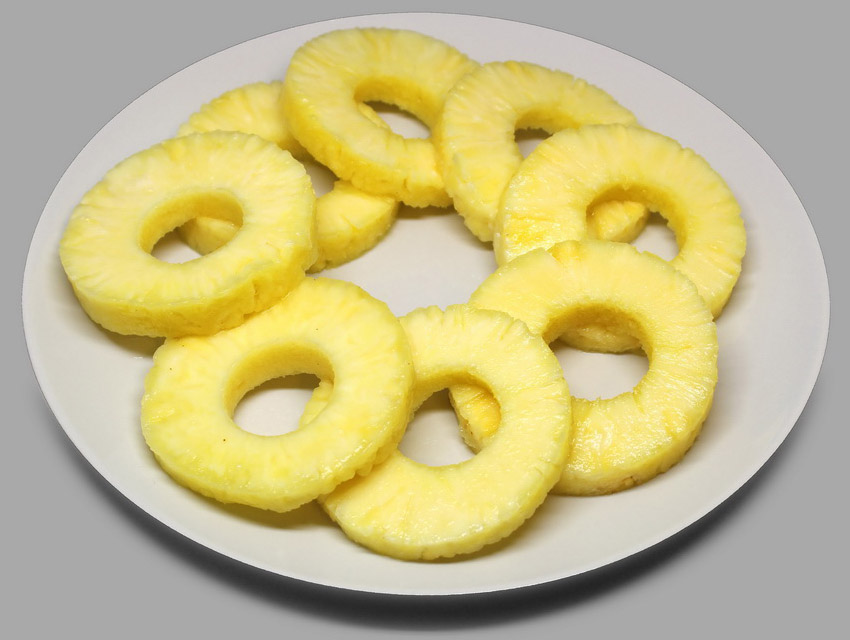

Supplement: Supplementary file 1 [file foods-10-01440-s001.zip › SupplementaryFiles/SuppBFoodImages/SubsetA/Palatable/tp0044.jpg]

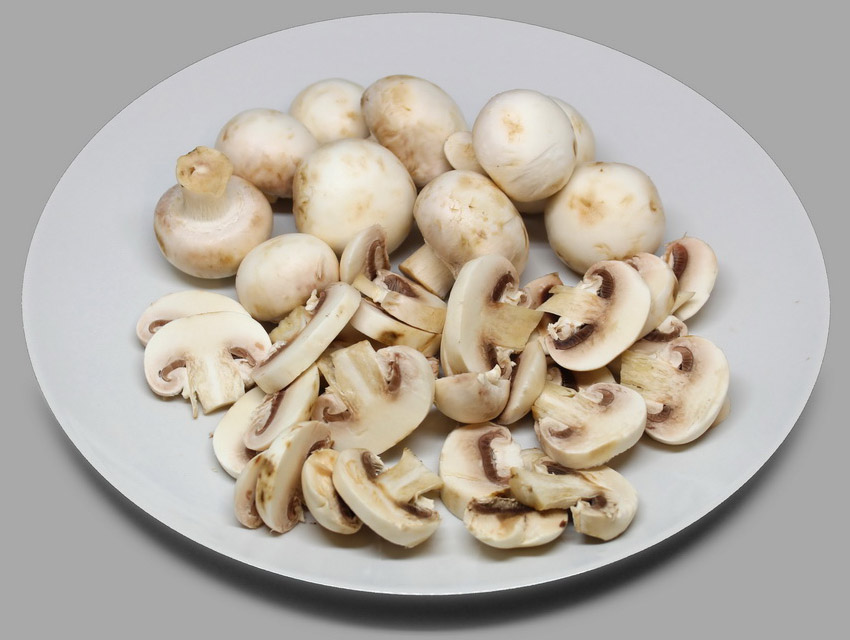

Supplement: Supplementary file 1 [file foods-10-01440-s001.zip › SupplementaryFiles/SuppBFoodImages/SubsetA/Palatable/tp0065.jpg]

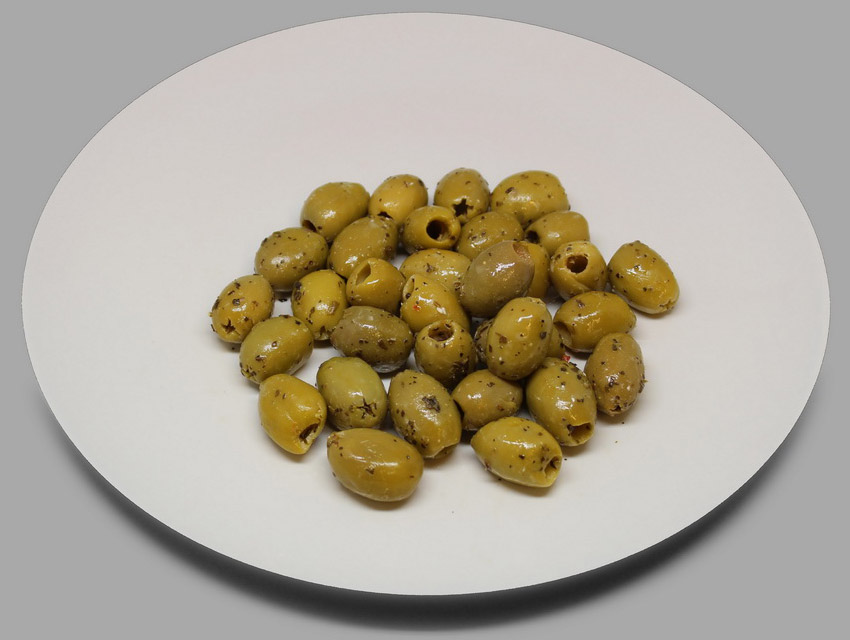

Supplement: Supplementary file 1 [file foods-10-01440-s001.zip › SupplementaryFiles/SuppBFoodImages/SubsetA/Palatable/tp0080.jpg]

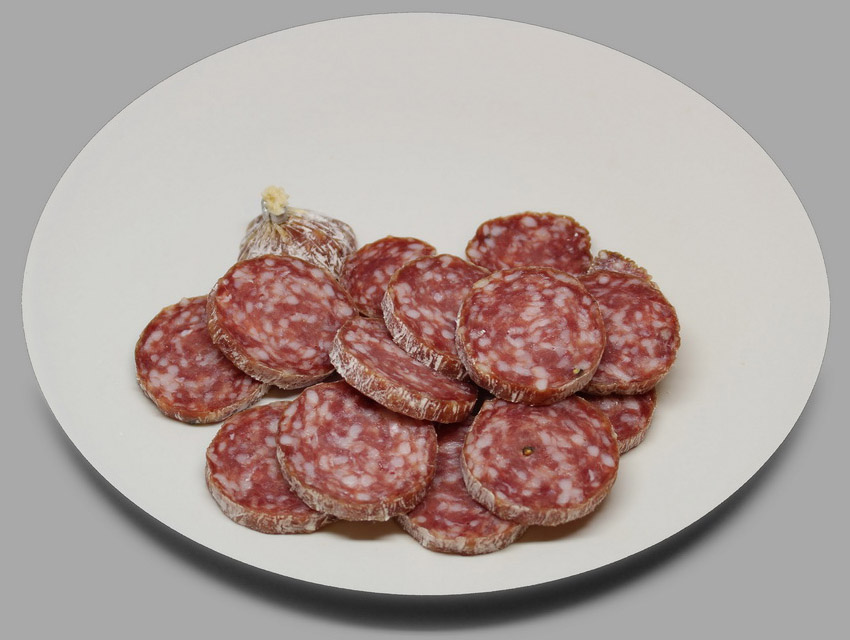

Supplement: Supplementary file 1 [file foods-10-01440-s001.zip › SupplementaryFiles/SuppBFoodImages/SubsetA/Palatable/tp0136.jpg]

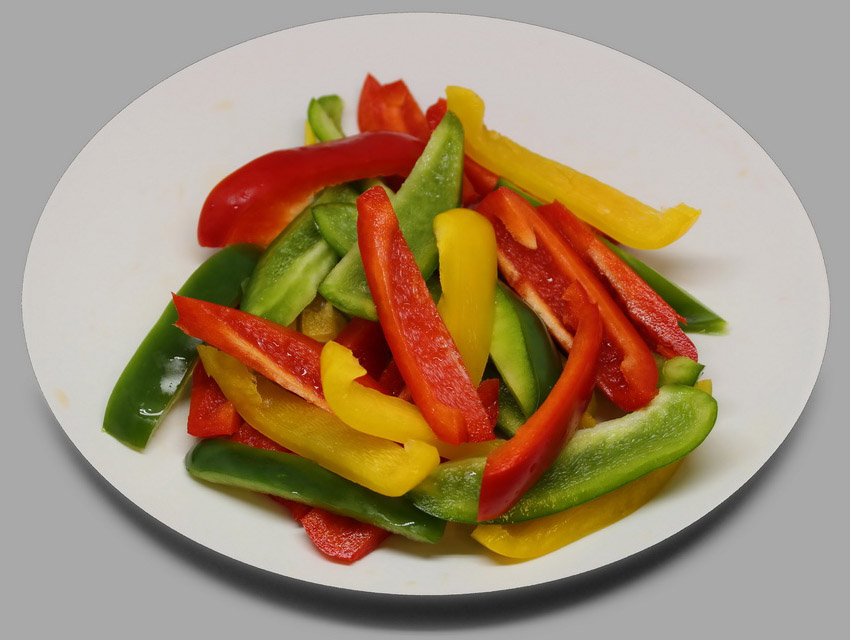

Supplement: Supplementary file 1 [file foods-10-01440-s001.zip › SupplementaryFiles/SuppBFoodImages/SubsetA/Palatable/tp0162.jpg]

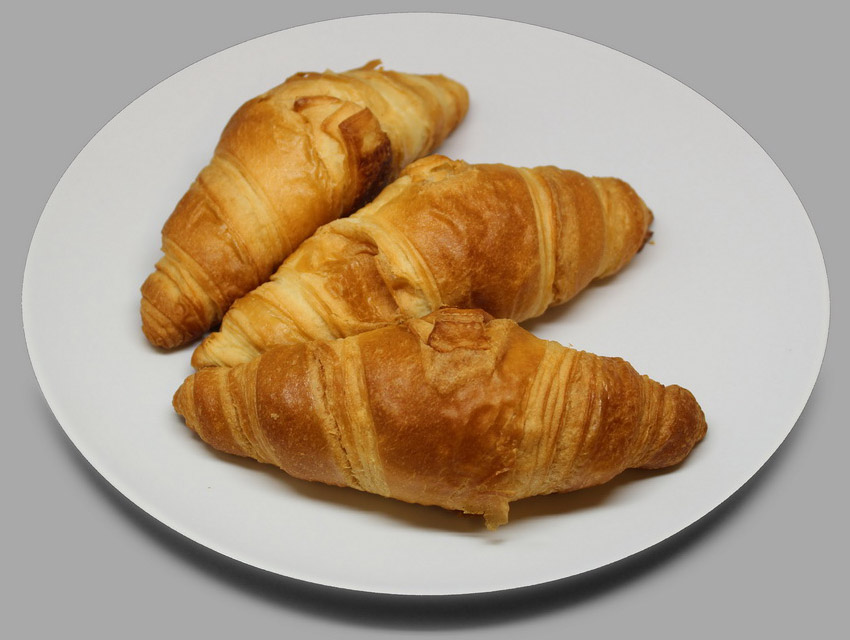

Supplement: Supplementary file 1 [file foods-10-01440-s001.zip › SupplementaryFiles/SuppBFoodImages/SubsetA/Palatable/tp0301.jpg]

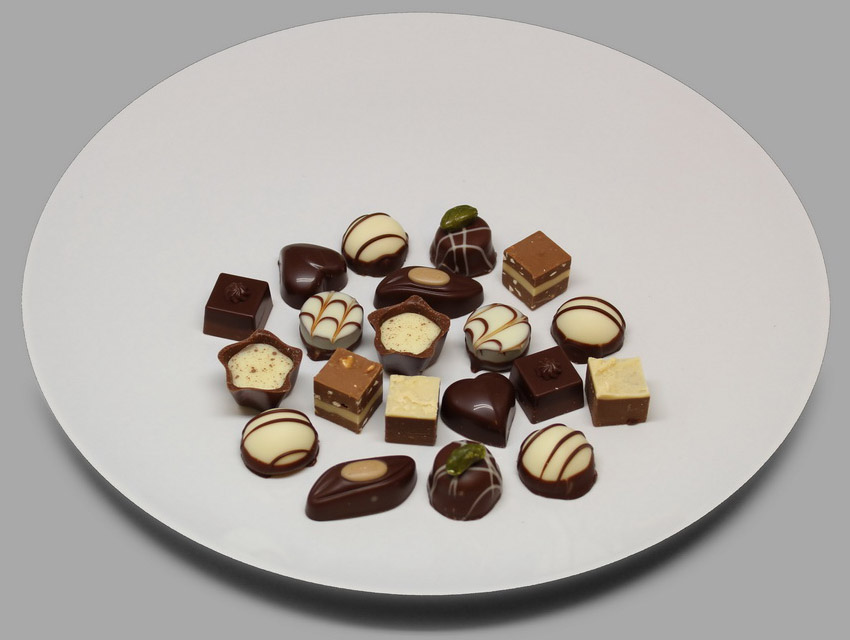

Supplement: Supplementary file 1 [file foods-10-01440-s001.zip › SupplementaryFiles/SuppBFoodImages/SubsetA/Palatable/tp0325.jpg]

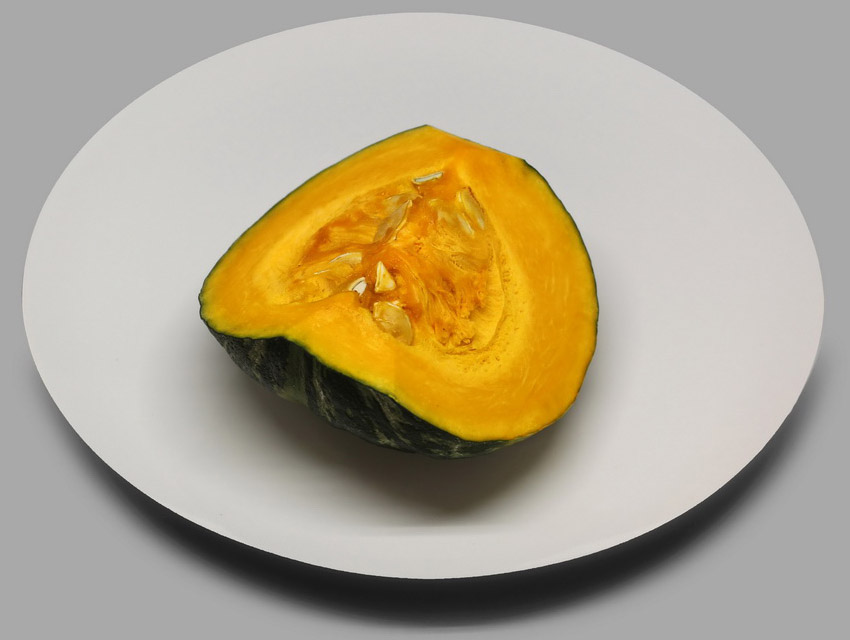

Supplement: Supplementary file 1 [file foods-10-01440-s001.zip › SupplementaryFiles/SuppBFoodImages/SubsetA/Palatable/tp0716.jpg]

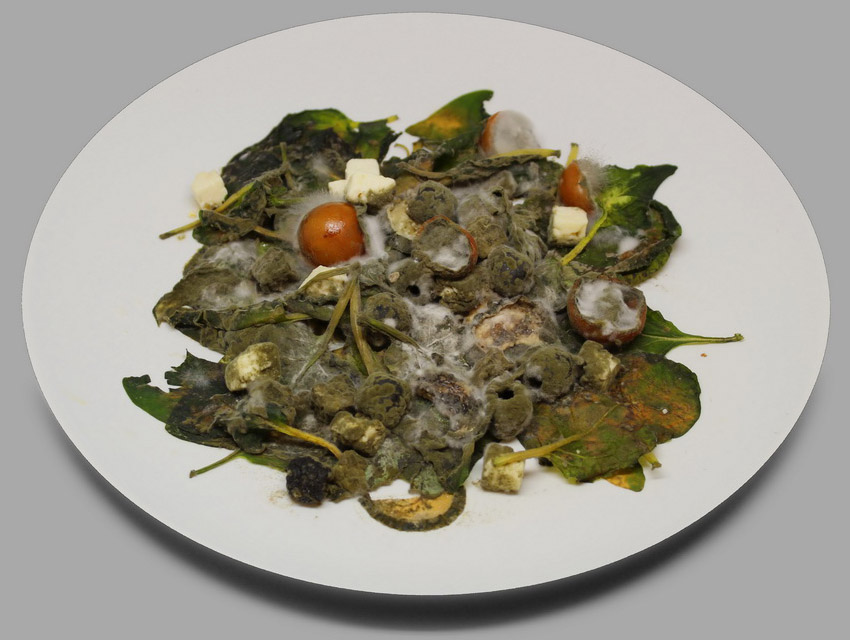

Supplement: Supplementary file 1 [file foods-10-01440-s001.zip › SupplementaryFiles/SuppBFoodImages/SubsetA/Unpalatable/tp0109.jpg]

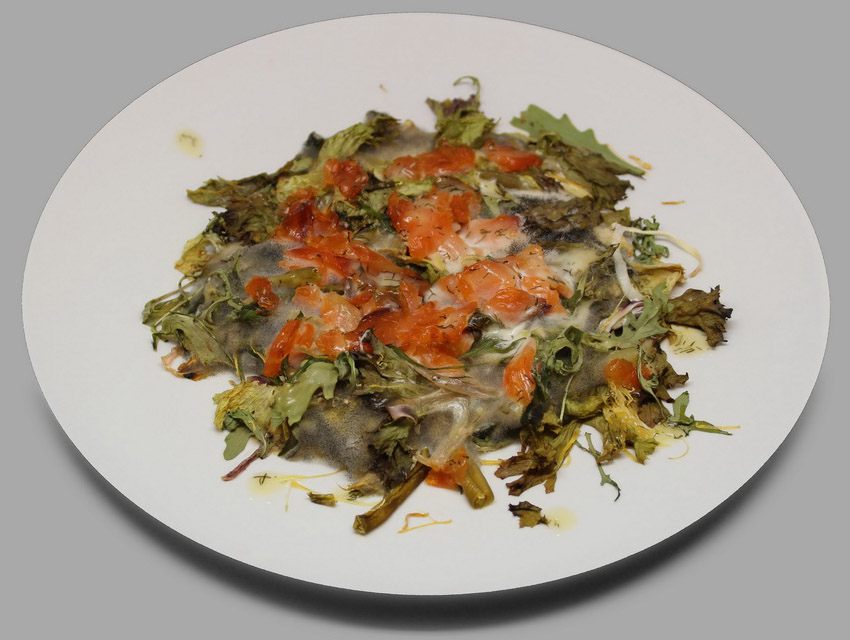

Supplement: Supplementary file 1 [file foods-10-01440-s001.zip › SupplementaryFiles/SuppBFoodImages/SubsetA/Unpalatable/tp0154.jpg]

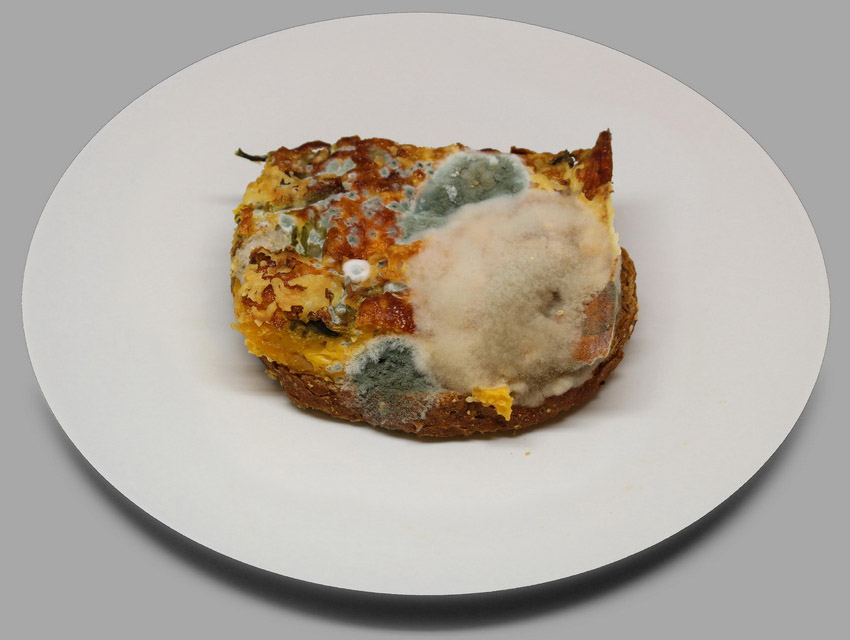

Supplement: Supplementary file 1 [file foods-10-01440-s001.zip › SupplementaryFiles/SuppBFoodImages/SubsetA/Unpalatable/tp0168.jpg]

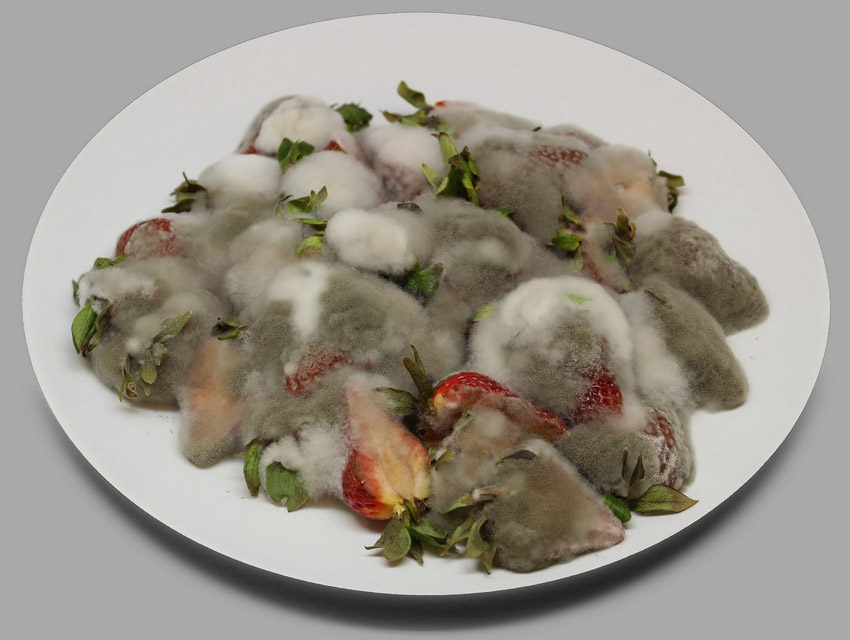

Supplement: Supplementary file 1 [file foods-10-01440-s001.zip › SupplementaryFiles/SuppBFoodImages/SubsetA/Unpalatable/tp0184.jpg]

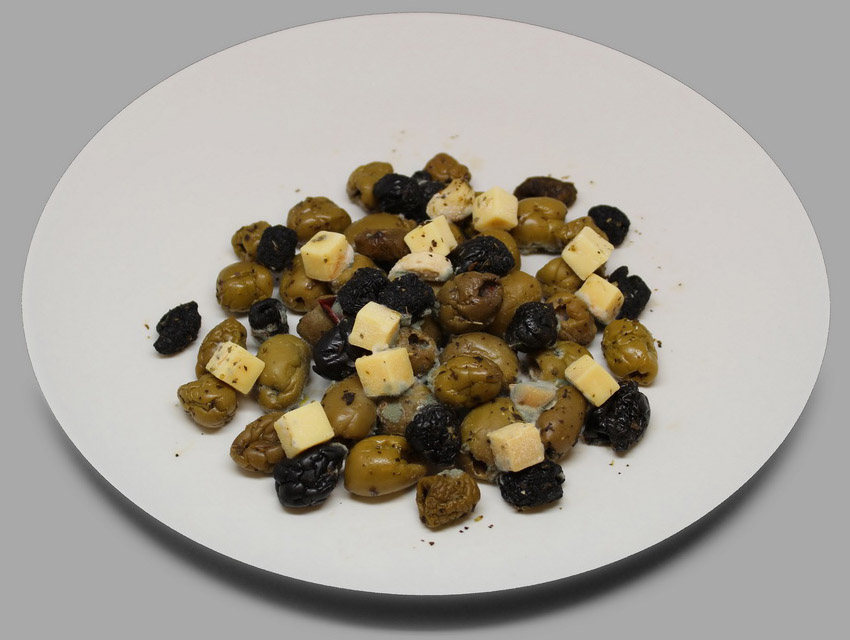

Supplement: Supplementary file 1 [file foods-10-01440-s001.zip › SupplementaryFiles/SuppBFoodImages/SubsetA/Unpalatable/tp0185.jpg]

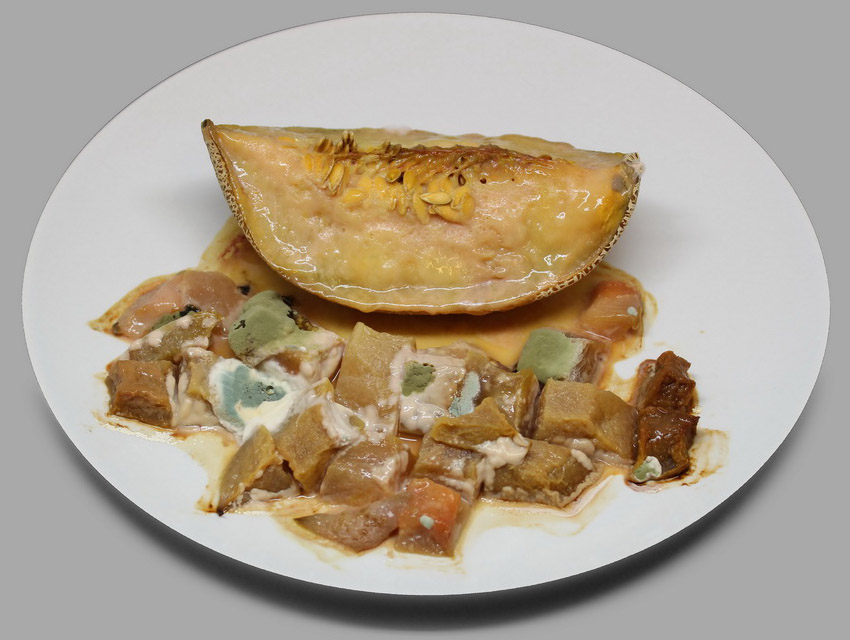

Supplement: Supplementary file 1 [file foods-10-01440-s001.zip › SupplementaryFiles/SuppBFoodImages/SubsetA/Unpalatable/tp0189.jpg]

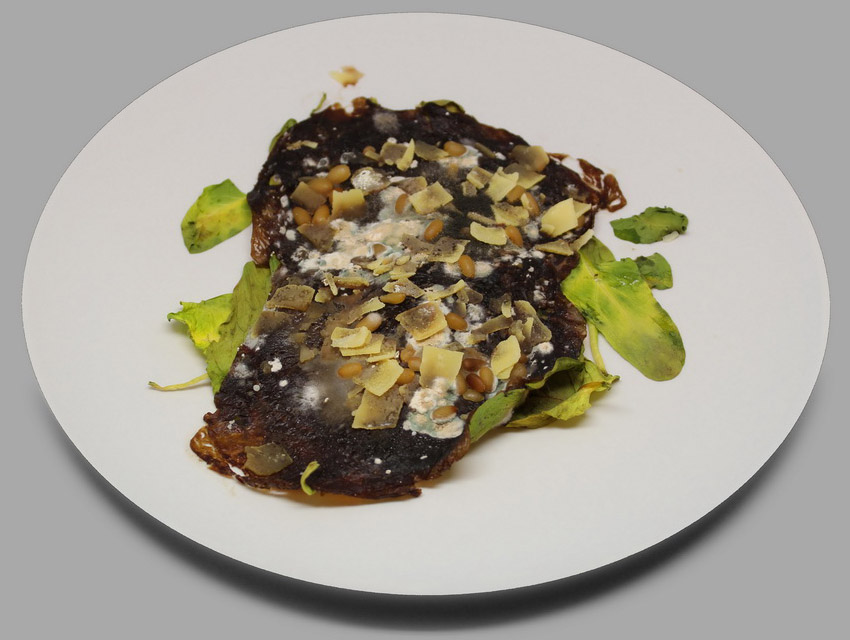

Supplement: Supplementary file 1 [file foods-10-01440-s001.zip › SupplementaryFiles/SuppBFoodImages/SubsetA/Unpalatable/tp0191.jpg]

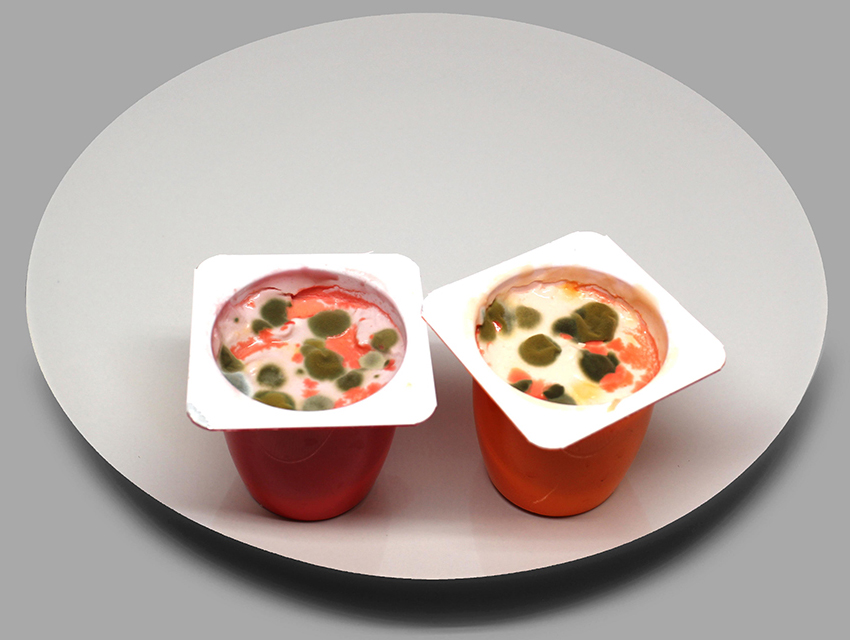

Supplement: Supplementary file 1 [file foods-10-01440-s001.zip › SupplementaryFiles/SuppBFoodImages/SubsetA/Unpalatable/tp0963.jpg]

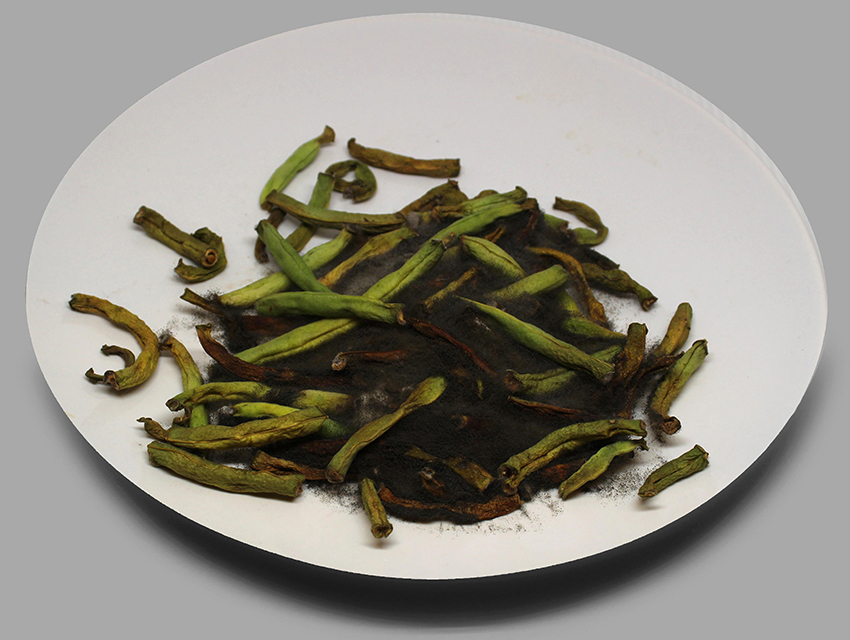

Supplement: Supplementary file 1 [file foods-10-01440-s001.zip › SupplementaryFiles/SuppBFoodImages/SubsetA/Unpalatable/tp0978.jpg]

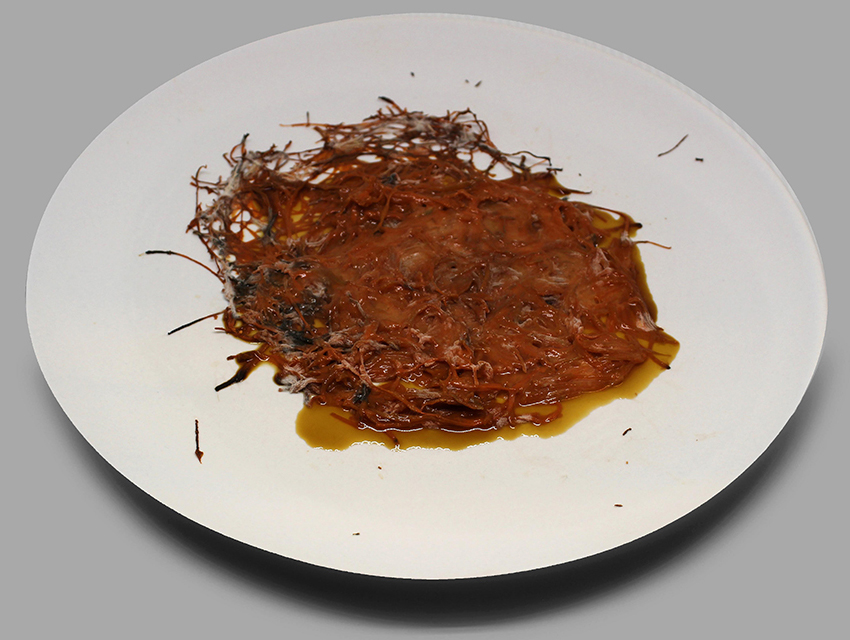

Supplement: Supplementary file 1 [file foods-10-01440-s001.zip › SupplementaryFiles/SuppBFoodImages/SubsetA/Unpalatable/tp0982.jpg]

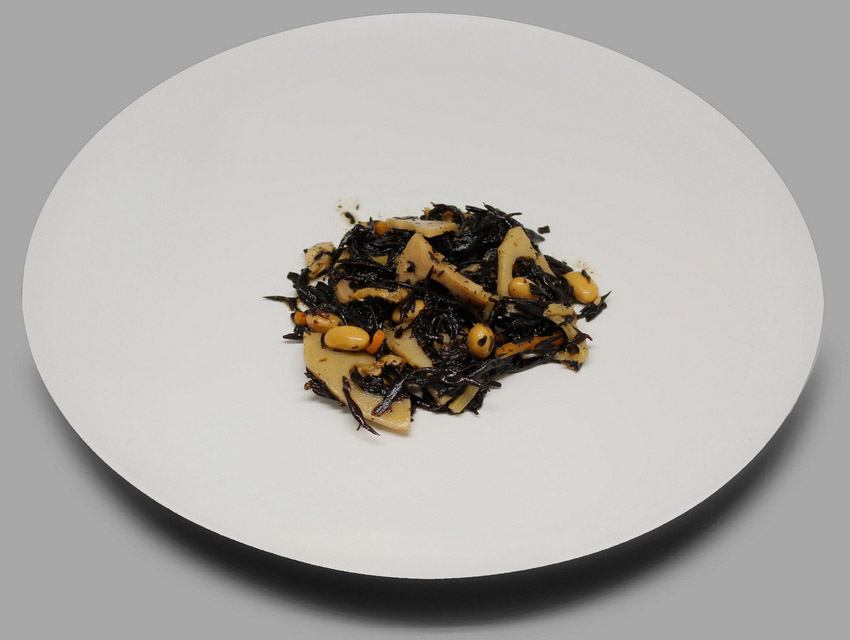

Supplement: Supplementary file 1 [file foods-10-01440-s001.zip › SupplementaryFiles/SuppBFoodImages/SubsetB/Asian/tp0372.jpg]

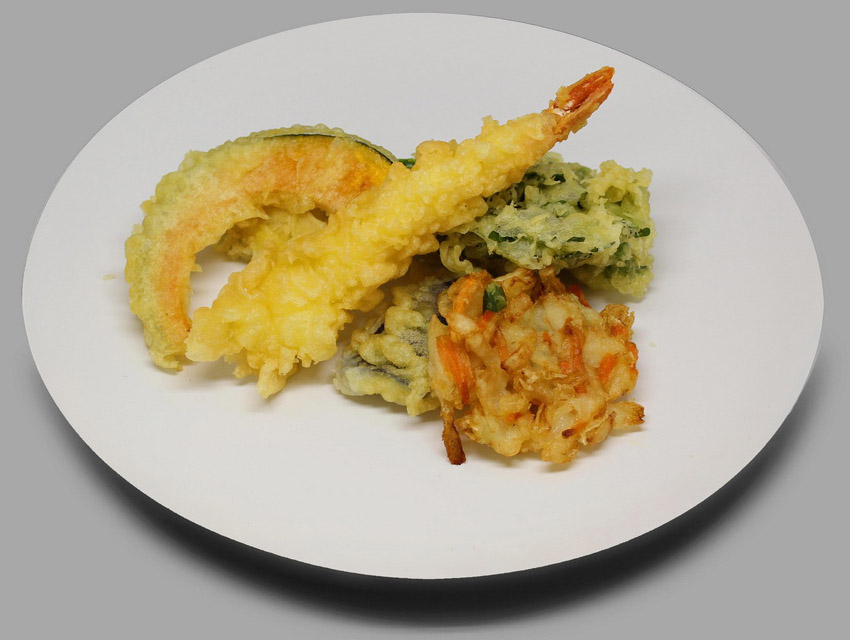

Supplement: Supplementary file 1 [file foods-10-01440-s001.zip › SupplementaryFiles/SuppBFoodImages/SubsetB/Asian/tp0393.jpg]

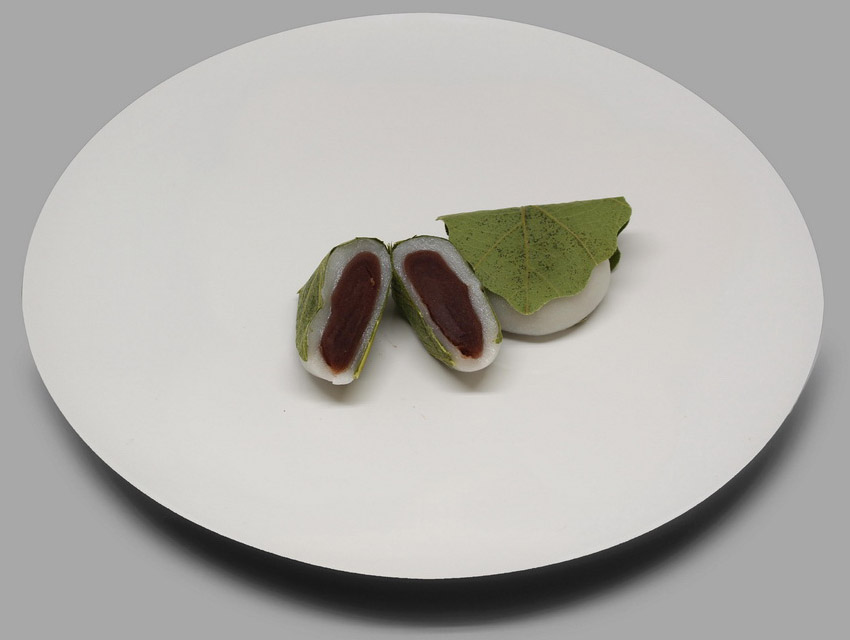

Supplement: Supplementary file 1 [file foods-10-01440-s001.zip › SupplementaryFiles/SuppBFoodImages/SubsetB/Asian/tp0418.jpg]

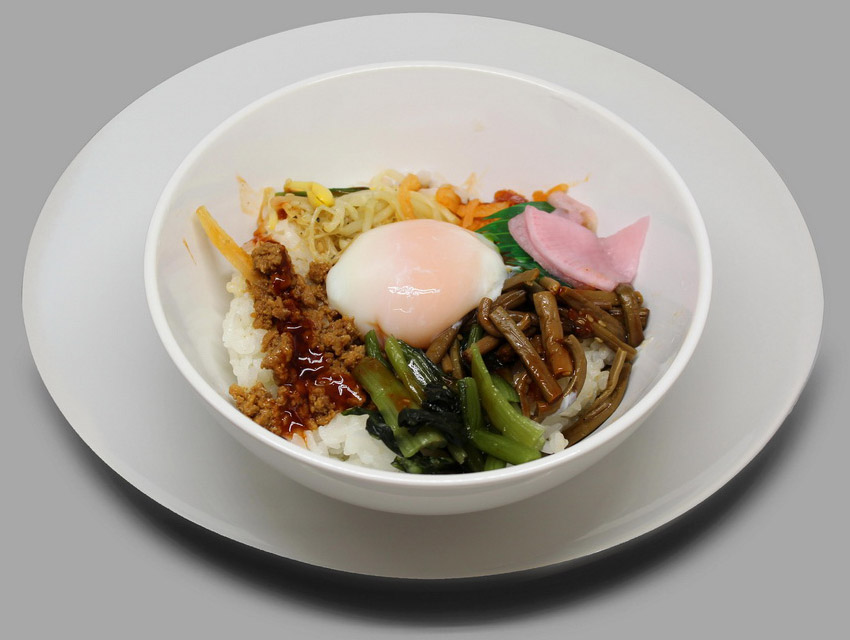

Supplement: Supplementary file 1 [file foods-10-01440-s001.zip › SupplementaryFiles/SuppBFoodImages/SubsetB/Asian/tp0437.jpg]

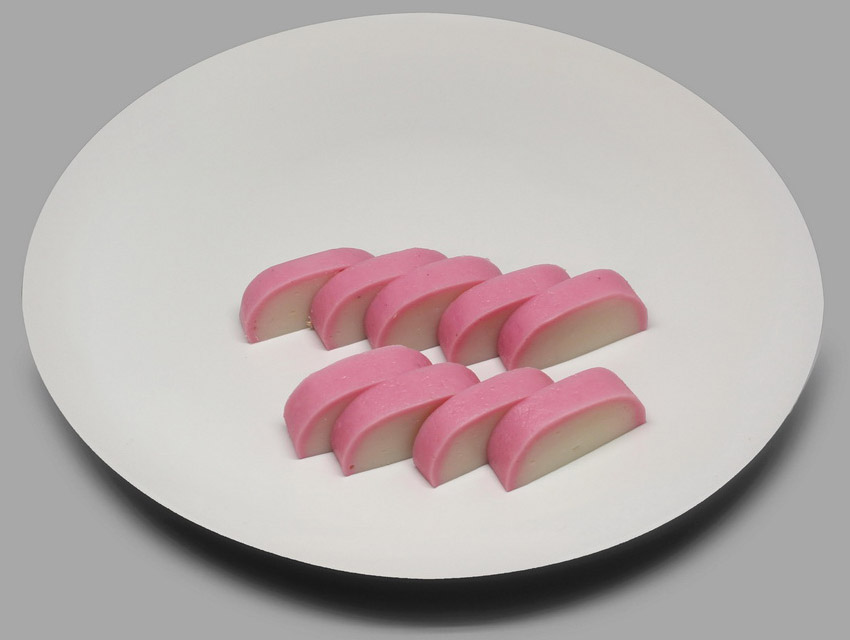

Supplement: Supplementary file 1 [file foods-10-01440-s001.zip › SupplementaryFiles/SuppBFoodImages/SubsetB/Asian/tp0467.jpg]

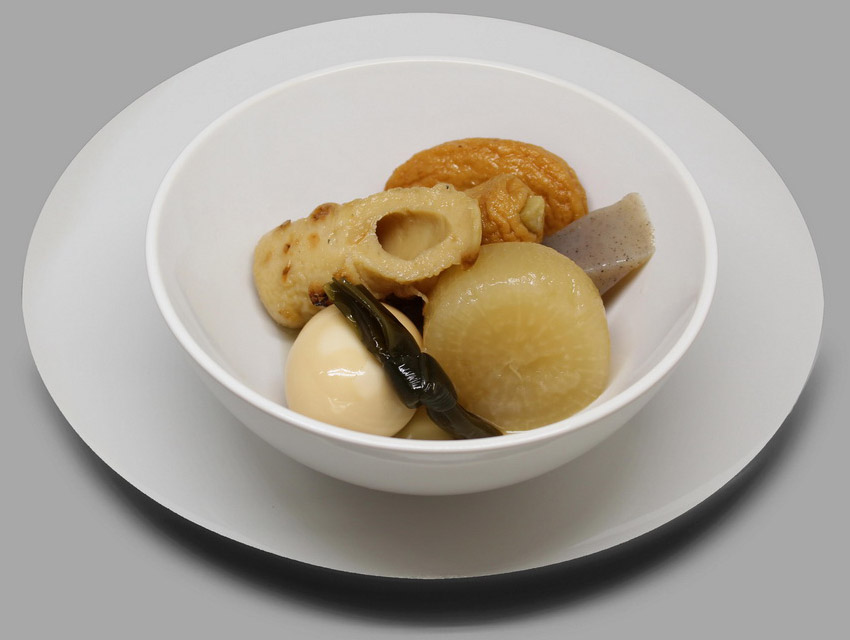

Supplement: Supplementary file 1 [file foods-10-01440-s001.zip › SupplementaryFiles/SuppBFoodImages/SubsetB/Asian/tp0476.jpg]

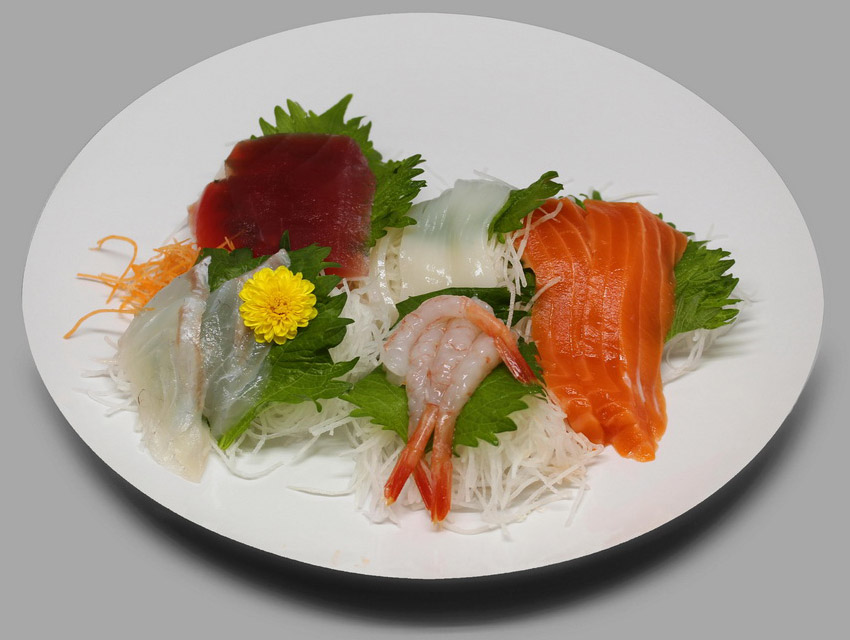

Supplement: Supplementary file 1 [file foods-10-01440-s001.zip › SupplementaryFiles/SuppBFoodImages/SubsetB/Asian/tp0481.jpg]

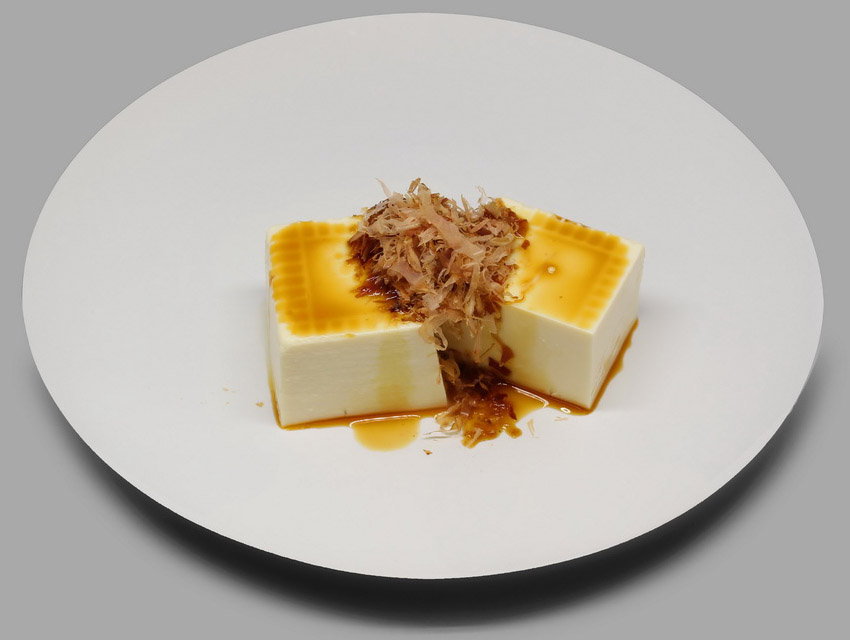

Supplement: Supplementary file 1 [file foods-10-01440-s001.zip › SupplementaryFiles/SuppBFoodImages/SubsetB/Asian/tp0517.jpg]

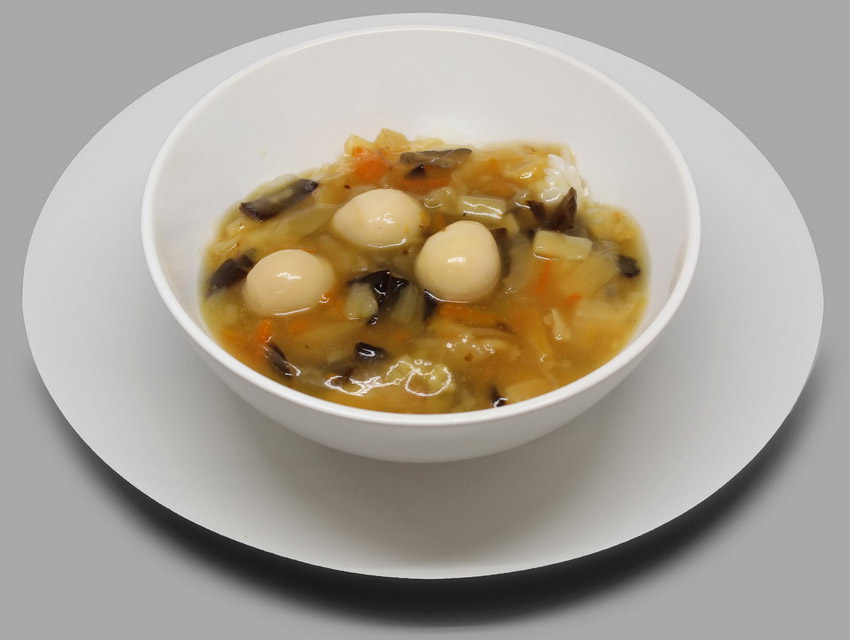

Supplement: Supplementary file 1 [file foods-10-01440-s001.zip › SupplementaryFiles/SuppBFoodImages/SubsetB/Asian/tp0531.jpg]

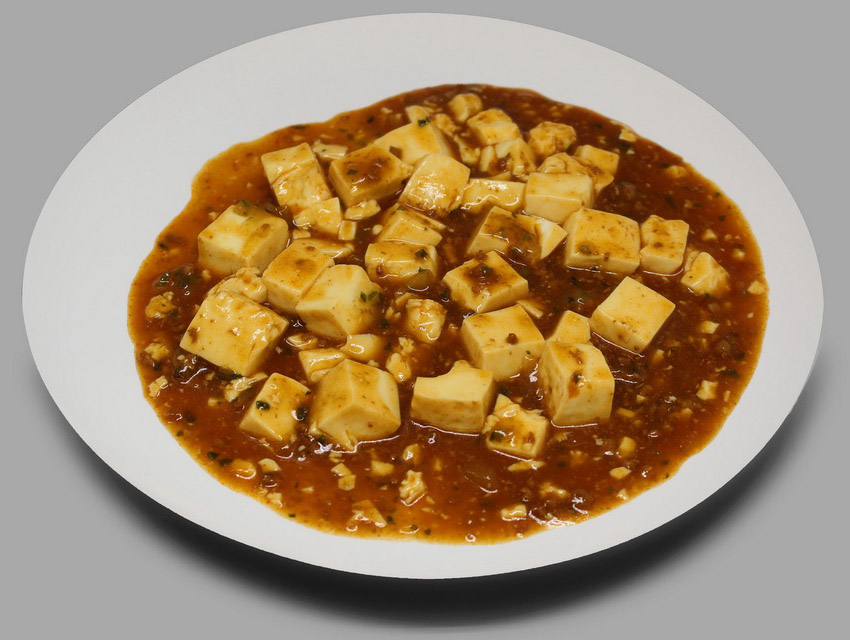

Supplement: Supplementary file 1 [file foods-10-01440-s001.zip › SupplementaryFiles/SuppBFoodImages/SubsetB/Asian/tp0551.jpg]

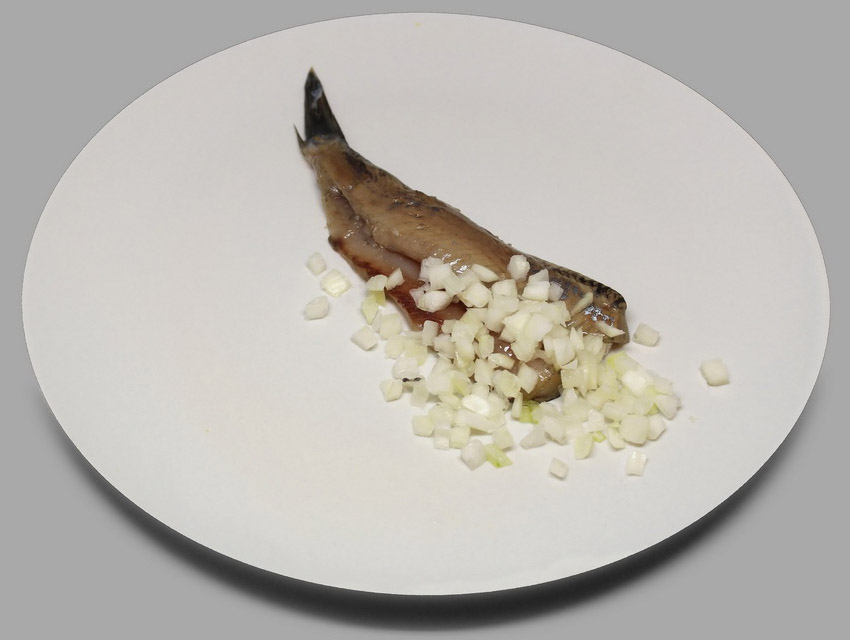

Supplement: Supplementary file 1 [file foods-10-01440-s001.zip › SupplementaryFiles/SuppBFoodImages/SubsetB/Dutch/tp0097.jpg]

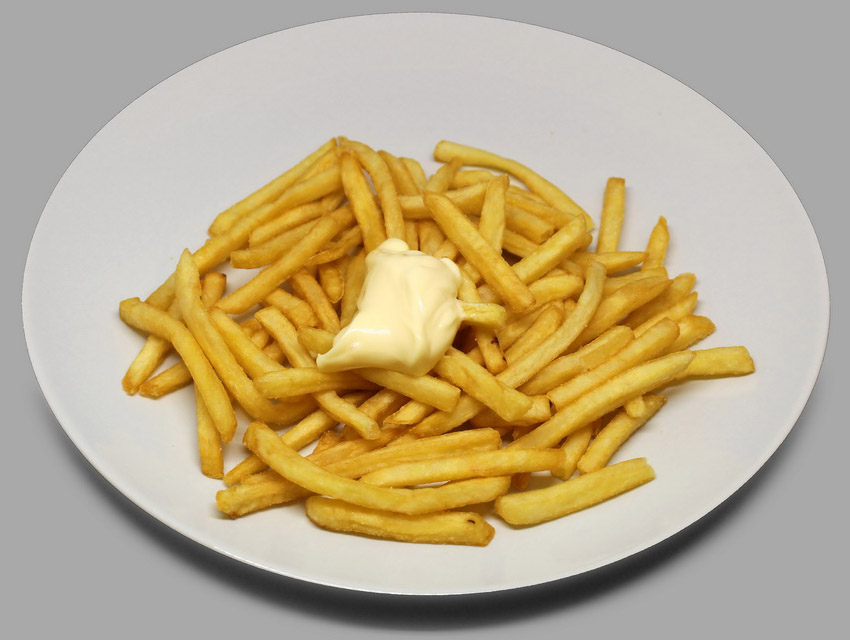

Supplement: Supplementary file 1 [file foods-10-01440-s001.zip › SupplementaryFiles/SuppBFoodImages/SubsetB/Dutch/tp0290.jpg]

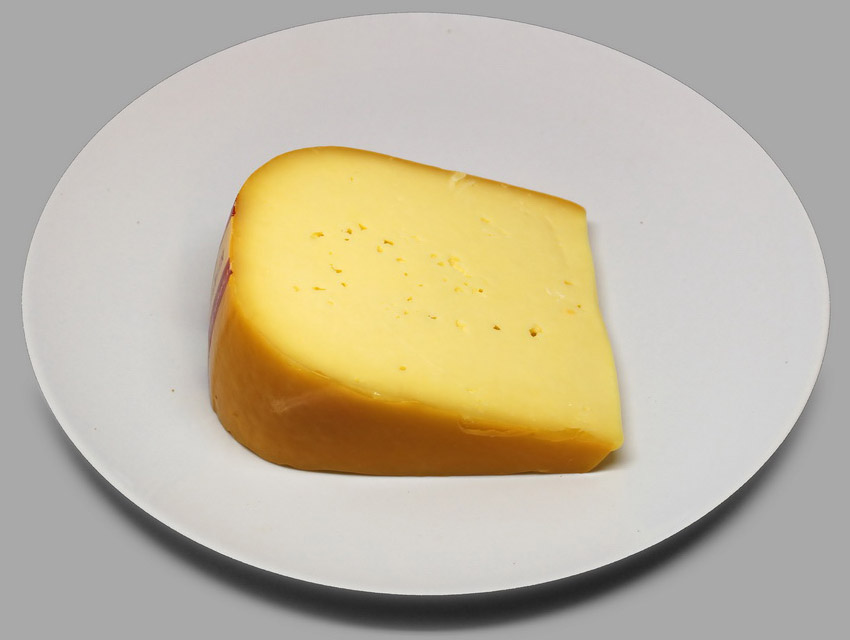

Supplement: Supplementary file 1 [file foods-10-01440-s001.zip › SupplementaryFiles/SuppBFoodImages/SubsetB/Dutch/tp0308.jpg]

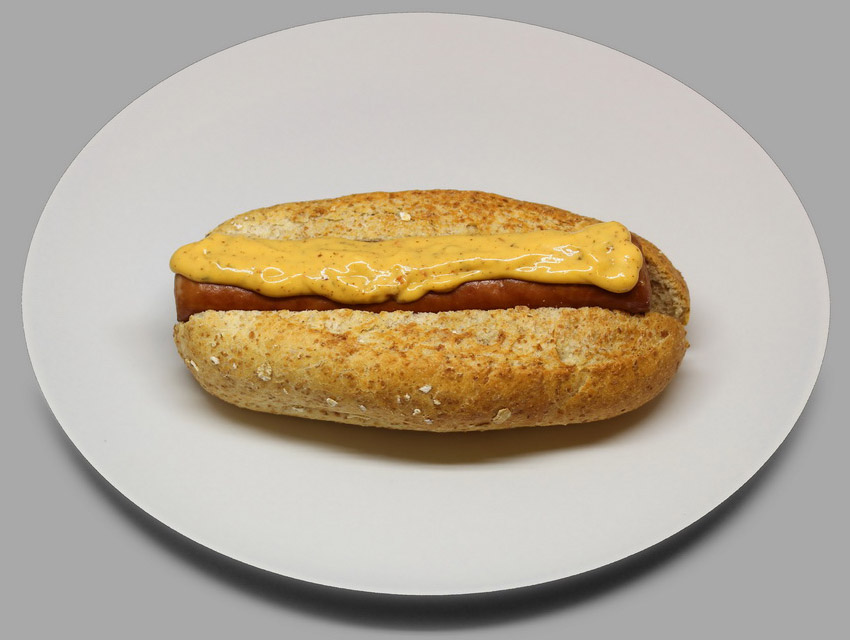

Supplement: Supplementary file 1 [file foods-10-01440-s001.zip › SupplementaryFiles/SuppBFoodImages/SubsetB/Dutch/tp0316.jpg]

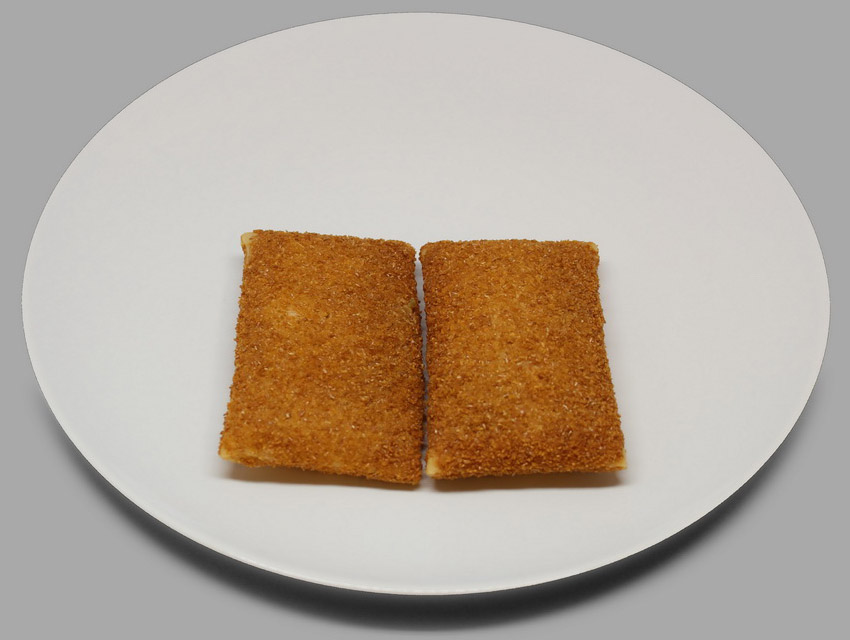

Supplement: Supplementary file 1 [file foods-10-01440-s001.zip › SupplementaryFiles/SuppBFoodImages/SubsetB/Dutch/tp0334.jpg]

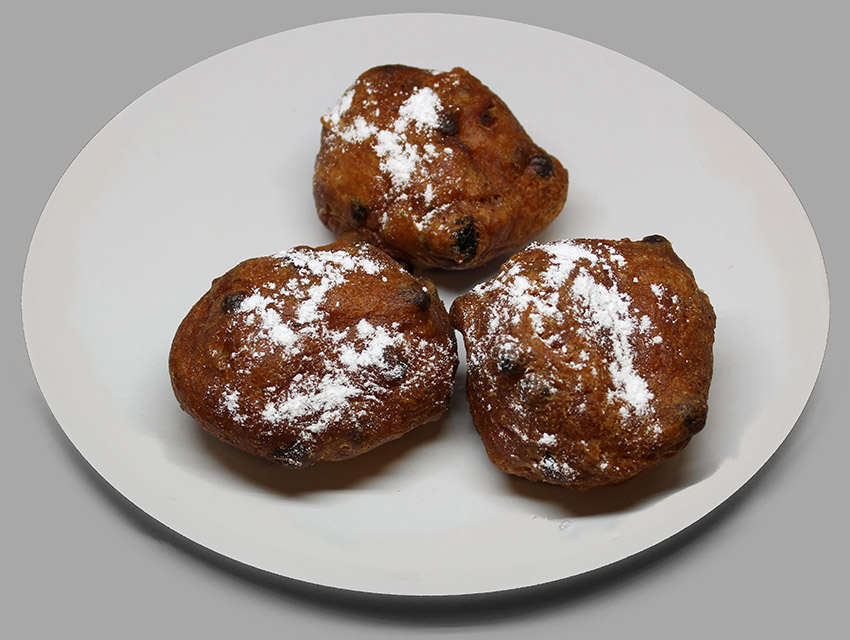

Supplement: Supplementary file 1 [file foods-10-01440-s001.zip › SupplementaryFiles/SuppBFoodImages/SubsetB/Dutch/tp0833.jpg]

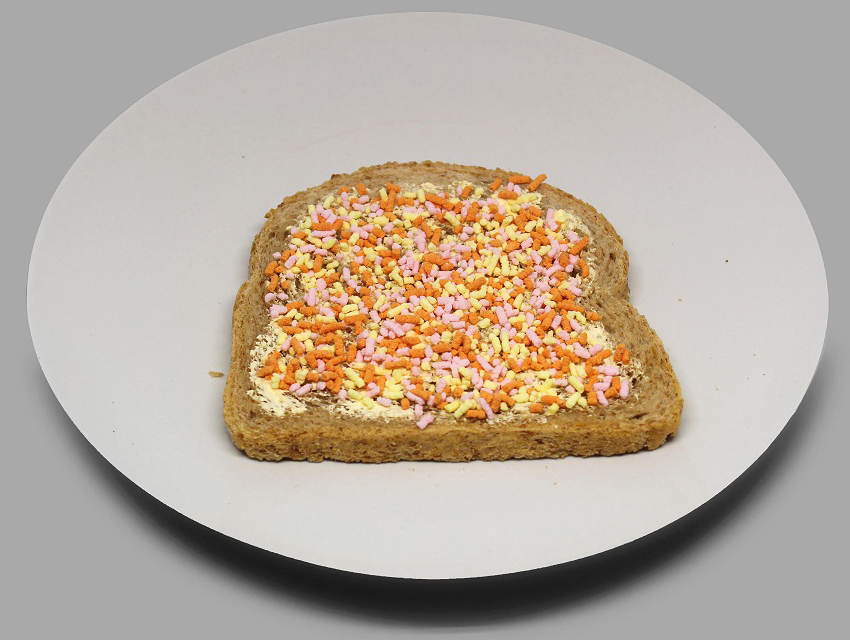

Supplement: Supplementary file 1 [file foods-10-01440-s001.zip › SupplementaryFiles/SuppBFoodImages/SubsetB/Dutch/tp0859.jpg]

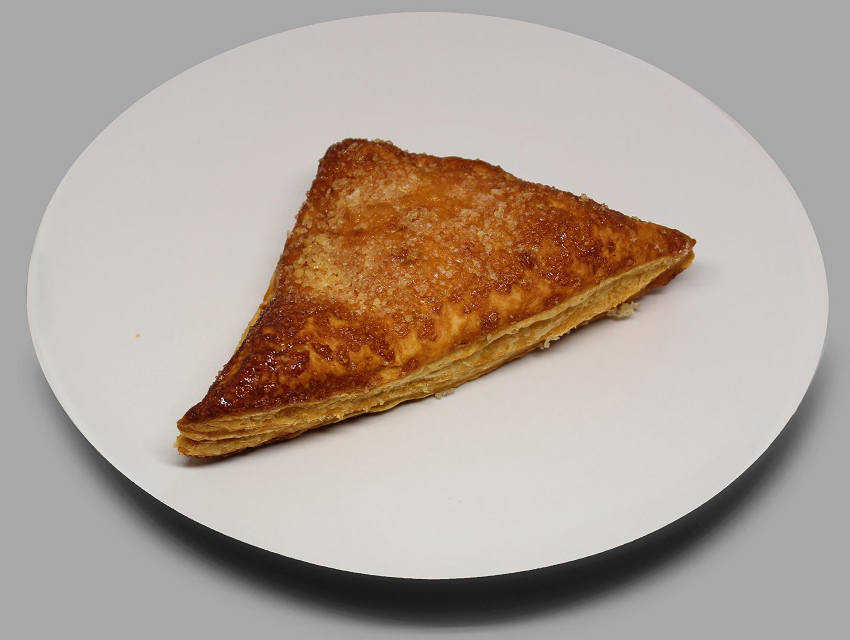

Supplement: Supplementary file 1 [file foods-10-01440-s001.zip › SupplementaryFiles/SuppBFoodImages/SubsetB/Dutch/tp0866.jpg]

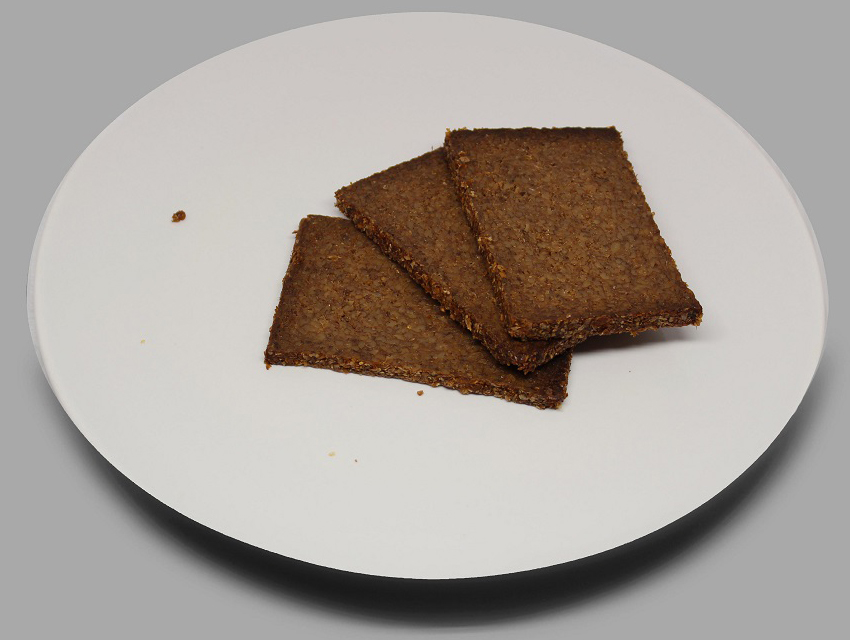

Supplement: Supplementary file 1 [file foods-10-01440-s001.zip › SupplementaryFiles/SuppBFoodImages/SubsetB/Dutch/tp0875.jpg]

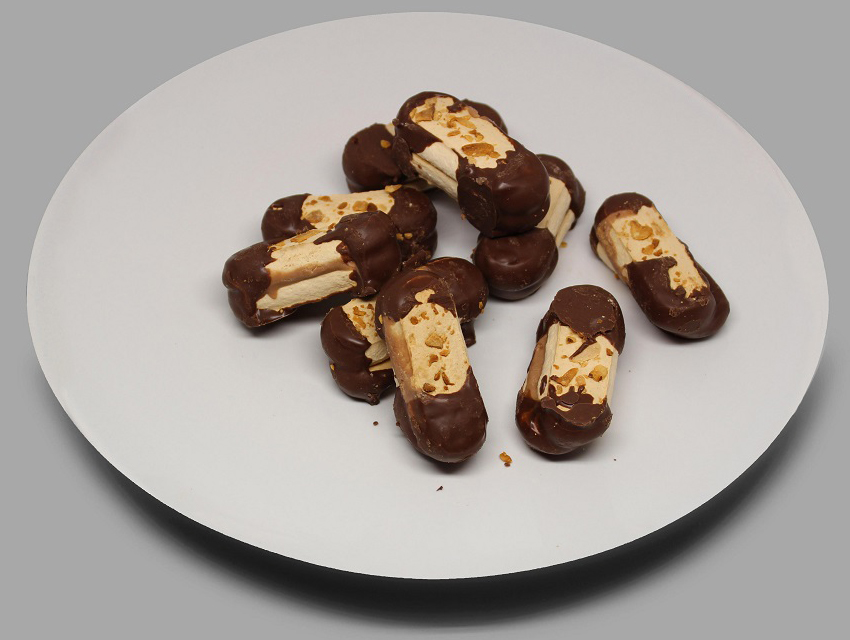

Supplement: Supplementary file 1 [file foods-10-01440-s001.zip › SupplementaryFiles/SuppBFoodImages/SubsetB/Dutch/tp0881.jpg]

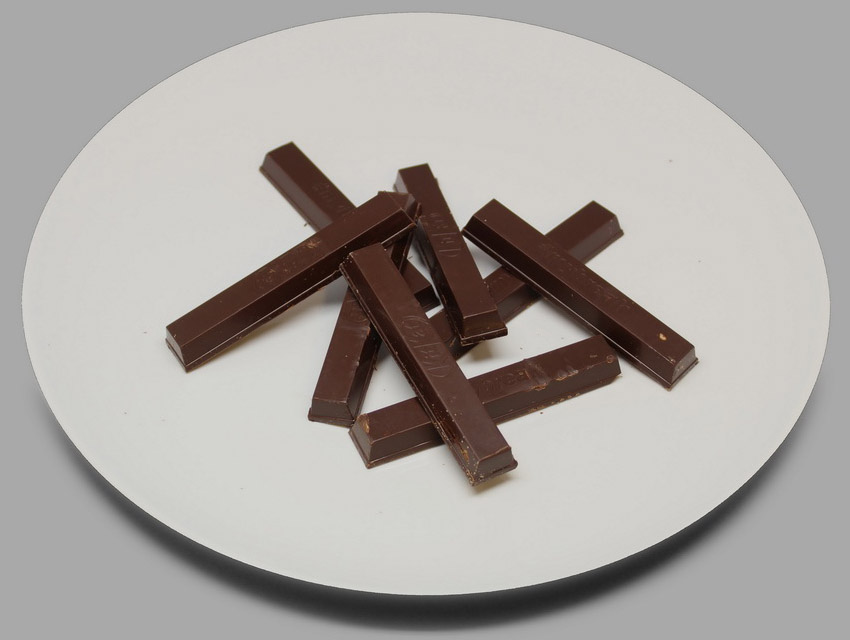

Supplement: Supplementary file 1 [file foods-10-01440-s001.zip › SupplementaryFiles/SuppBFoodImages/SubsetB/Palatable/tp0015.jpg]

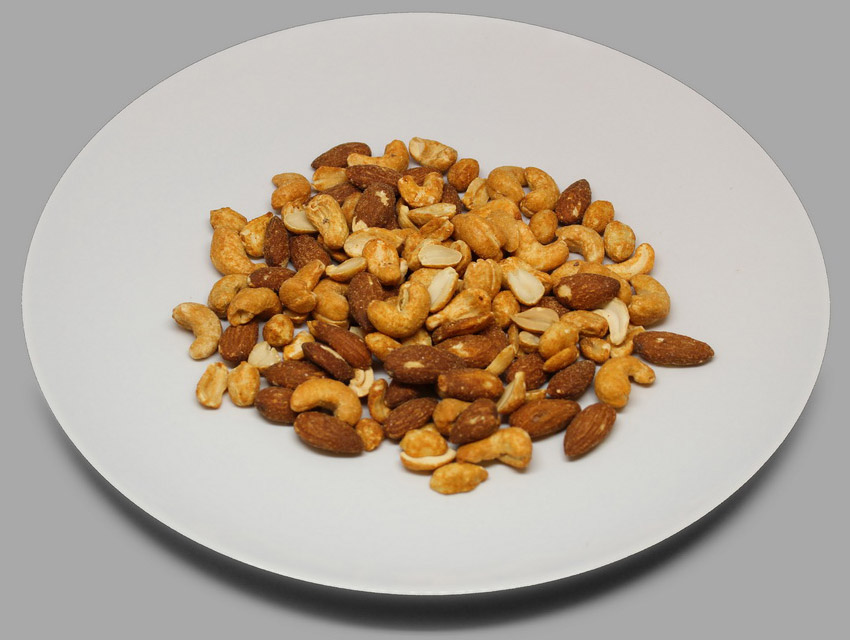

Supplement: Supplementary file 1 [file foods-10-01440-s001.zip › SupplementaryFiles/SuppBFoodImages/SubsetB/Palatable/tp0042.jpg]

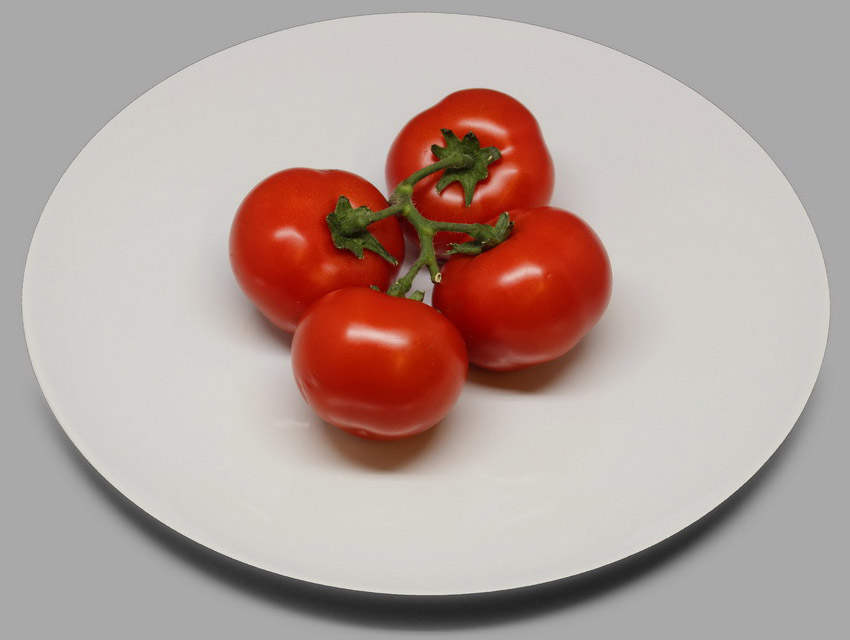

Supplement: Supplementary file 1 [file foods-10-01440-s001.zip › SupplementaryFiles/SuppBFoodImages/SubsetB/Palatable/tp0054.jpg]

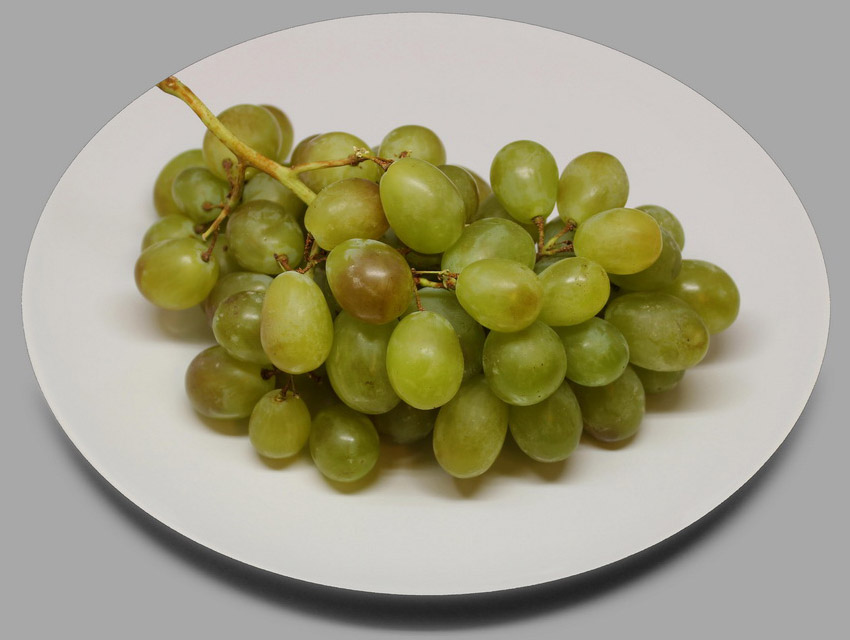

Supplement: Supplementary file 1 [file foods-10-01440-s001.zip › SupplementaryFiles/SuppBFoodImages/SubsetB/Palatable/tp0058.jpg]

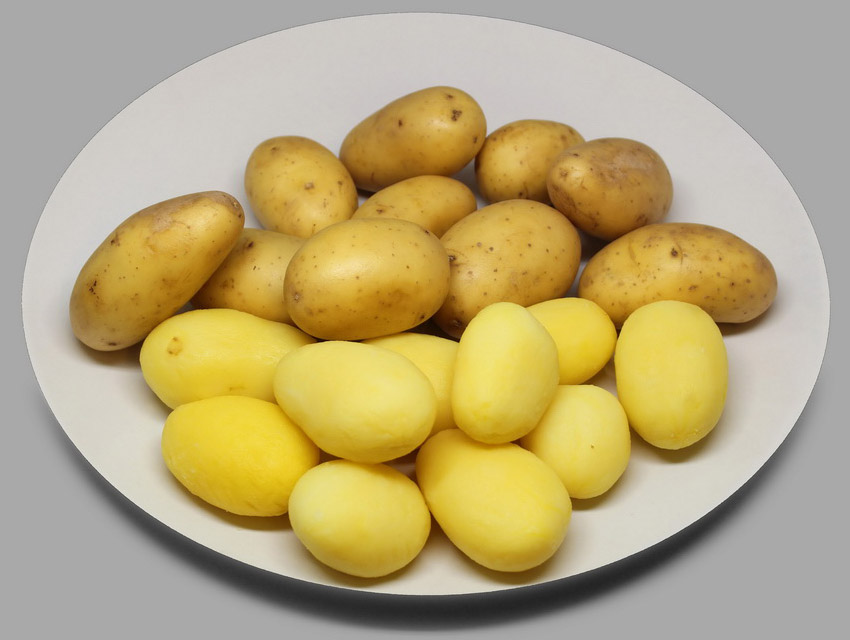

Supplement: Supplementary file 1 [file foods-10-01440-s001.zip › SupplementaryFiles/SuppBFoodImages/SubsetB/Palatable/tp0077.jpg]

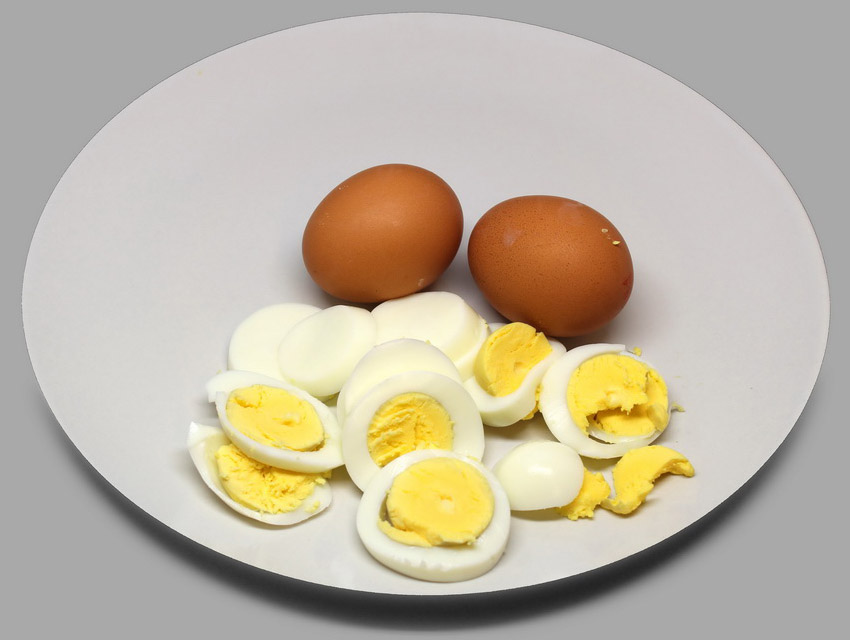

Supplement: Supplementary file 1 [file foods-10-01440-s001.zip › SupplementaryFiles/SuppBFoodImages/SubsetB/Palatable/tp0093.jpg]

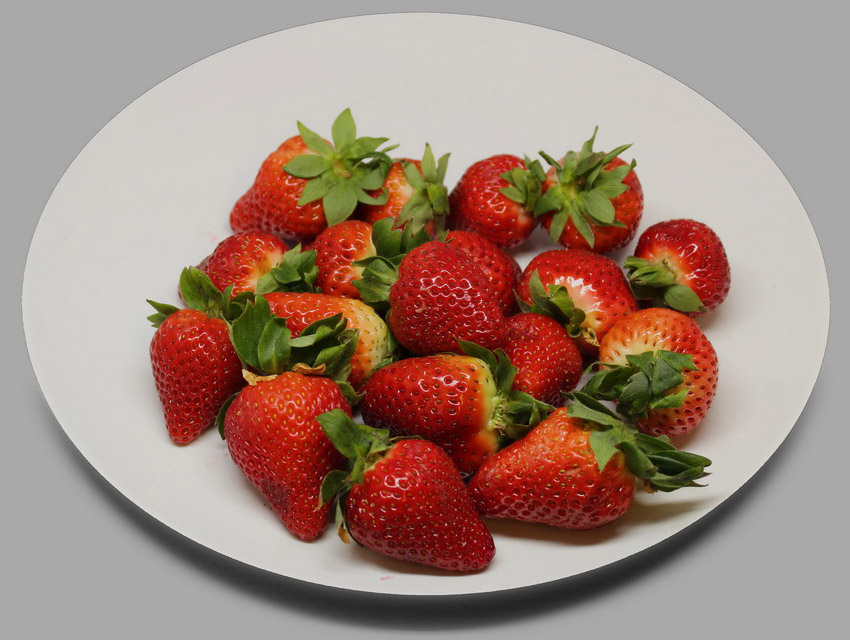

Supplement: Supplementary file 1 [file foods-10-01440-s001.zip › SupplementaryFiles/SuppBFoodImages/SubsetB/Palatable/tp0146.jpg]

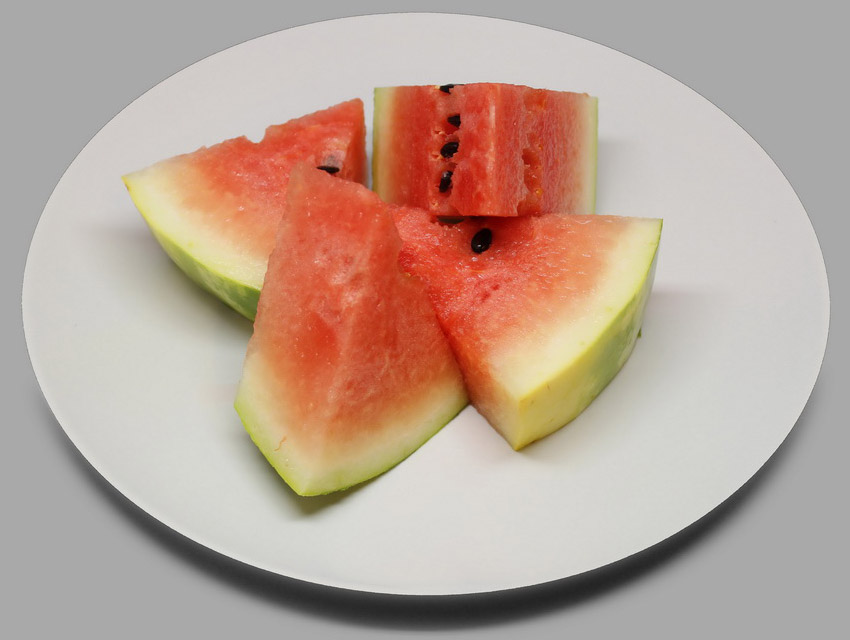

Supplement: Supplementary file 1 [file foods-10-01440-s001.zip › SupplementaryFiles/SuppBFoodImages/SubsetB/Palatable/tp0187.jpg]

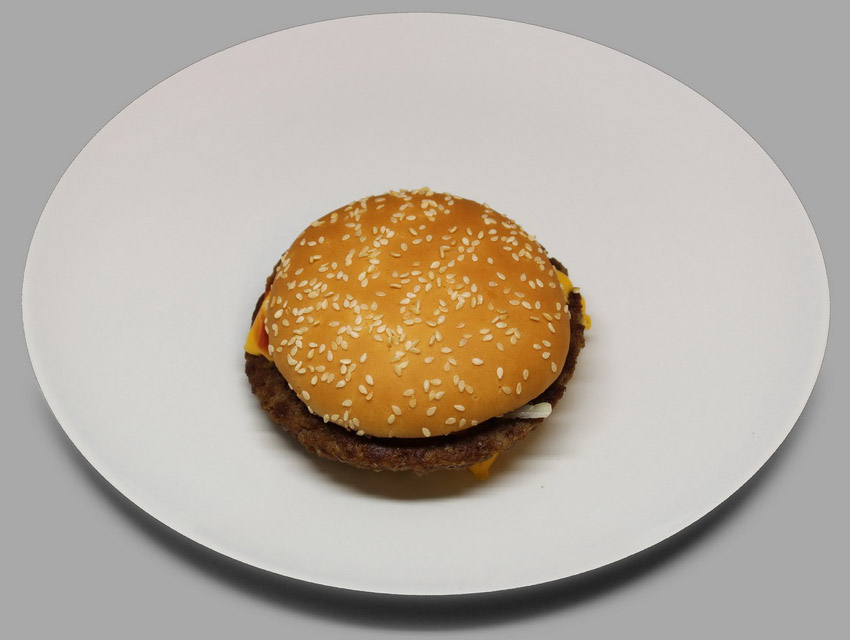

Supplement: Supplementary file 1 [file foods-10-01440-s001.zip › SupplementaryFiles/SuppBFoodImages/SubsetB/Palatable/tp0292.jpg]

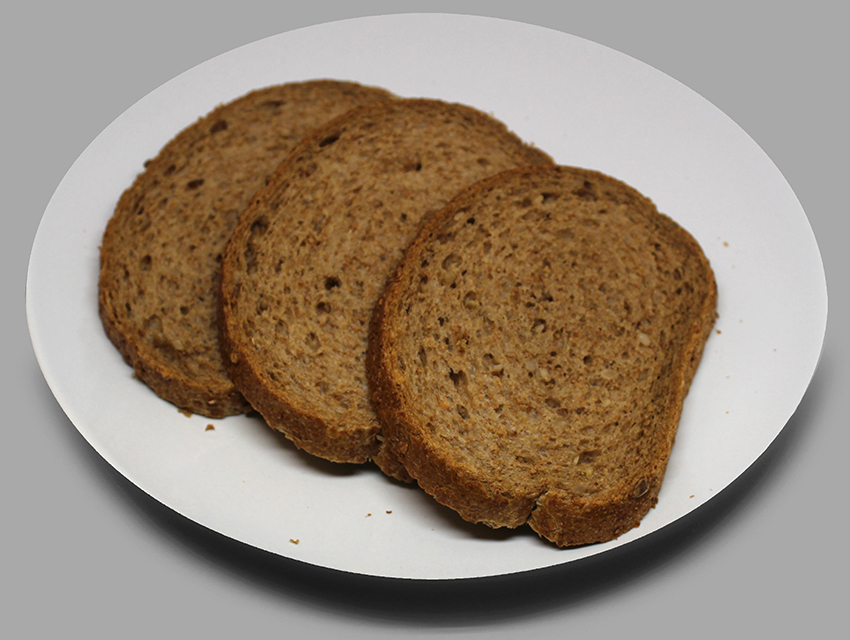

Supplement: Supplementary file 1 [file foods-10-01440-s001.zip › SupplementaryFiles/SuppBFoodImages/SubsetB/Palatable/tp0935.jpg]

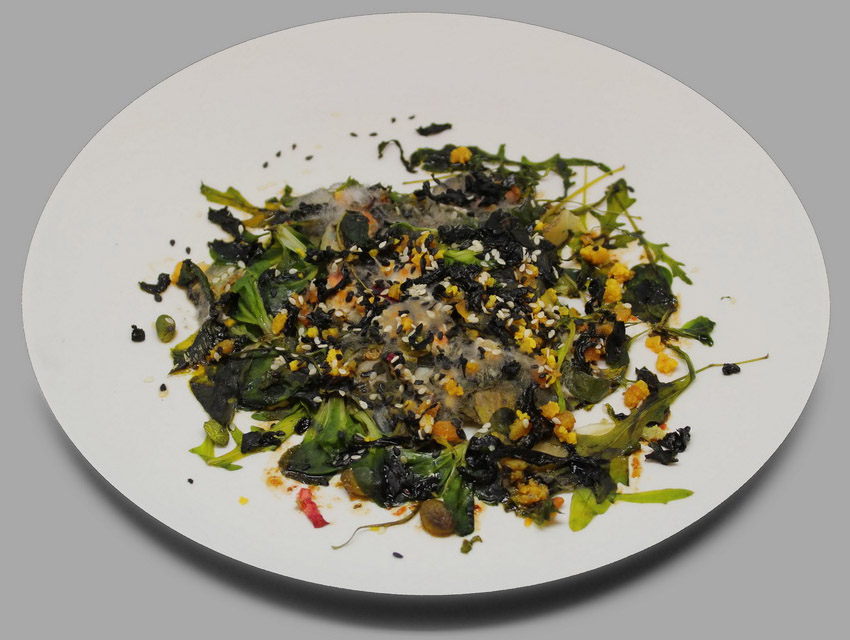

Supplement: Supplementary file 1 [file foods-10-01440-s001.zip › SupplementaryFiles/SuppBFoodImages/SubsetB/Unpalatable/tp0153.jpg]

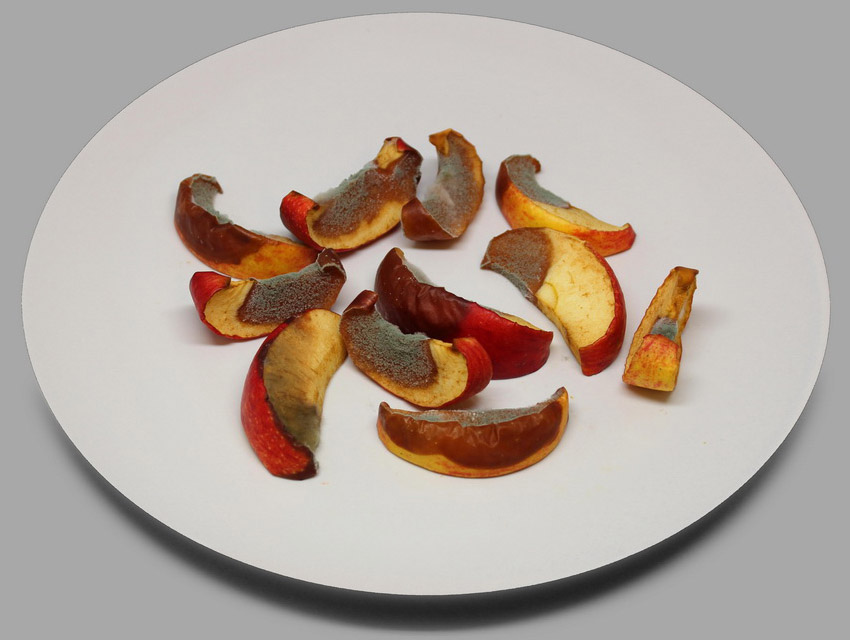

Supplement: Supplementary file 1 [file foods-10-01440-s001.zip › SupplementaryFiles/SuppBFoodImages/SubsetB/Unpalatable/tp0166.jpg]

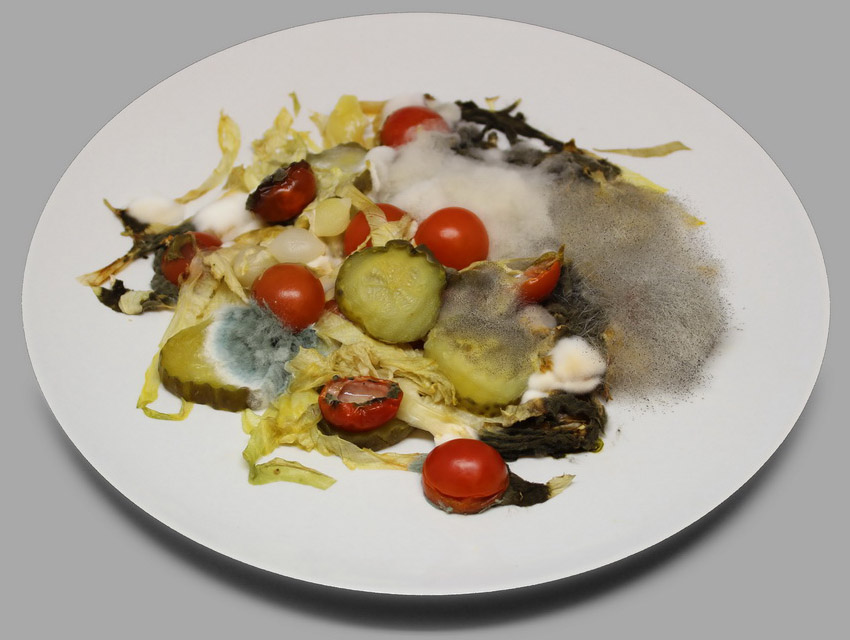

Supplement: Supplementary file 1 [file foods-10-01440-s001.zip › SupplementaryFiles/SuppBFoodImages/SubsetB/Unpalatable/tp0167.jpg]

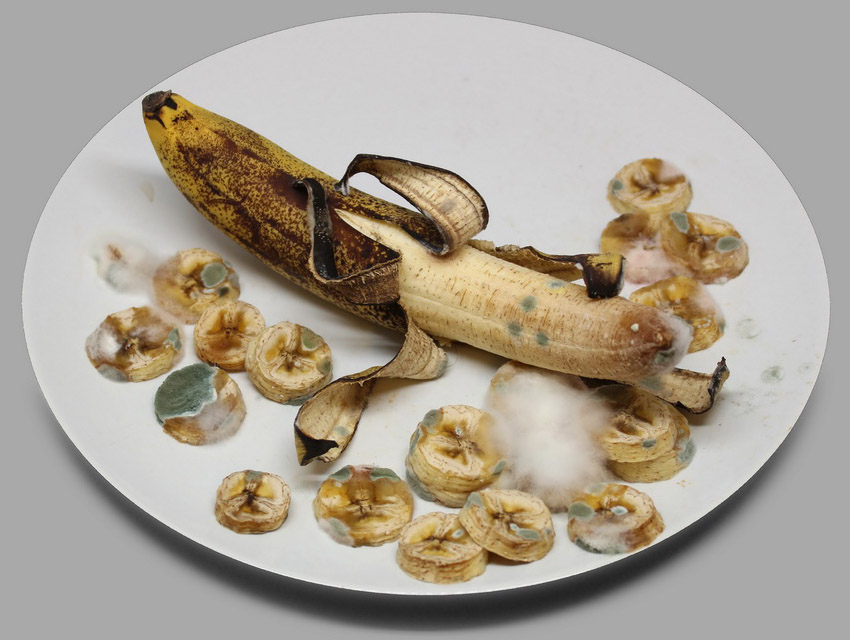

Supplement: Supplementary file 1 [file foods-10-01440-s001.zip › SupplementaryFiles/SuppBFoodImages/SubsetB/Unpalatable/tp0190.jpg]

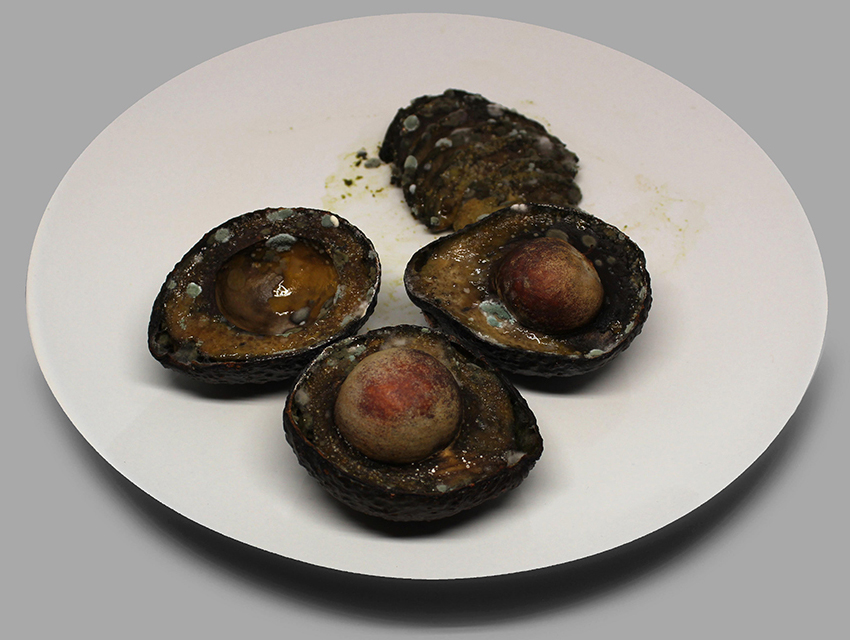

Supplement: Supplementary file 1 [file foods-10-01440-s001.zip › SupplementaryFiles/SuppBFoodImages/SubsetB/Unpalatable/tp0954.jpg]

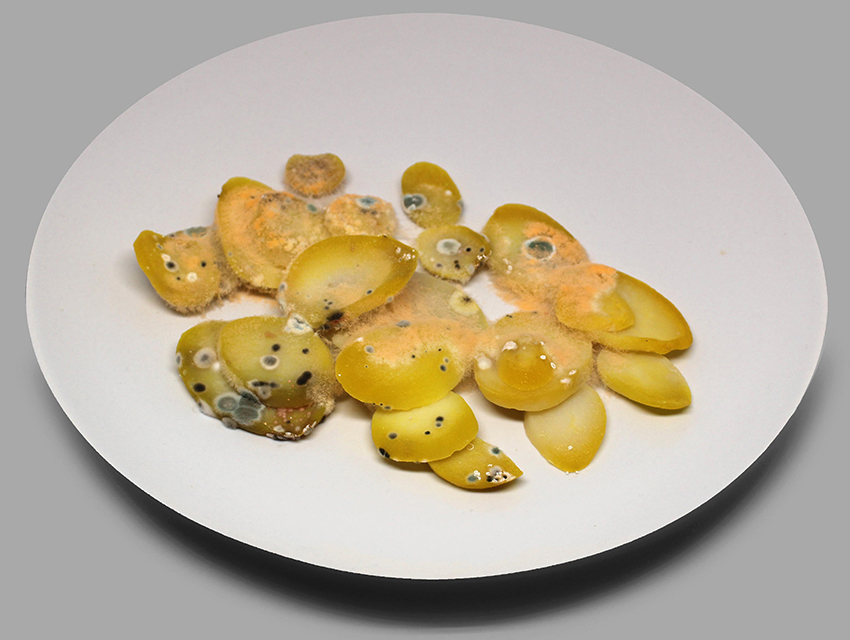

Supplement: Supplementary file 1 [file foods-10-01440-s001.zip › SupplementaryFiles/SuppBFoodImages/SubsetB/Unpalatable/tp0955.jpg]

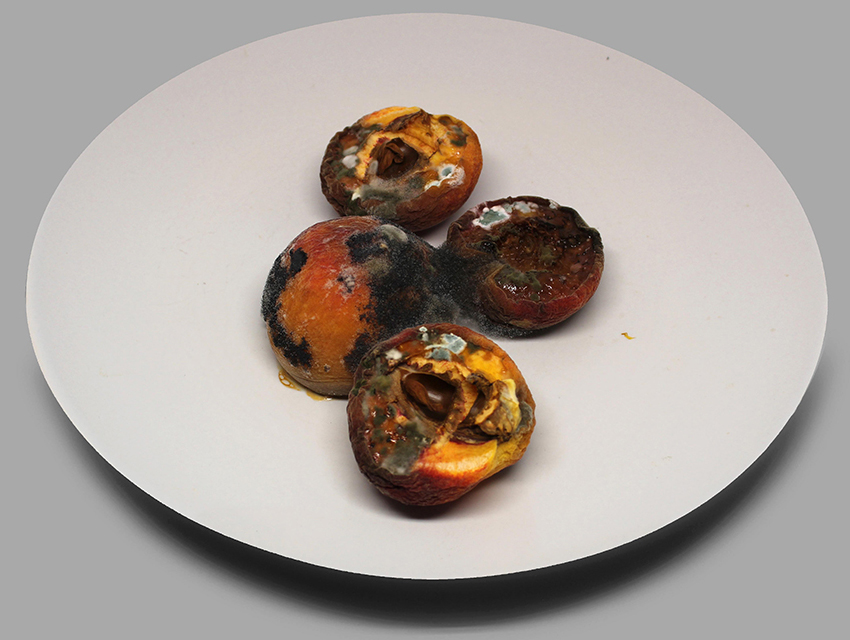

Supplement: Supplementary file 1 [file foods-10-01440-s001.zip › SupplementaryFiles/SuppBFoodImages/SubsetB/Unpalatable/tp0957.jpg]

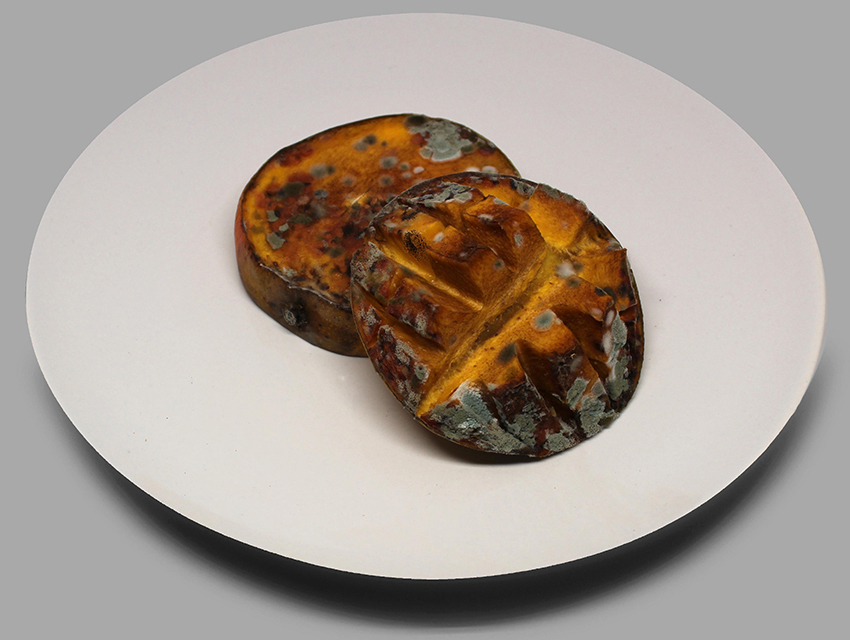

Supplement: Supplementary file 1 [file foods-10-01440-s001.zip › SupplementaryFiles/SuppBFoodImages/SubsetB/Unpalatable/tp0959.jpg]

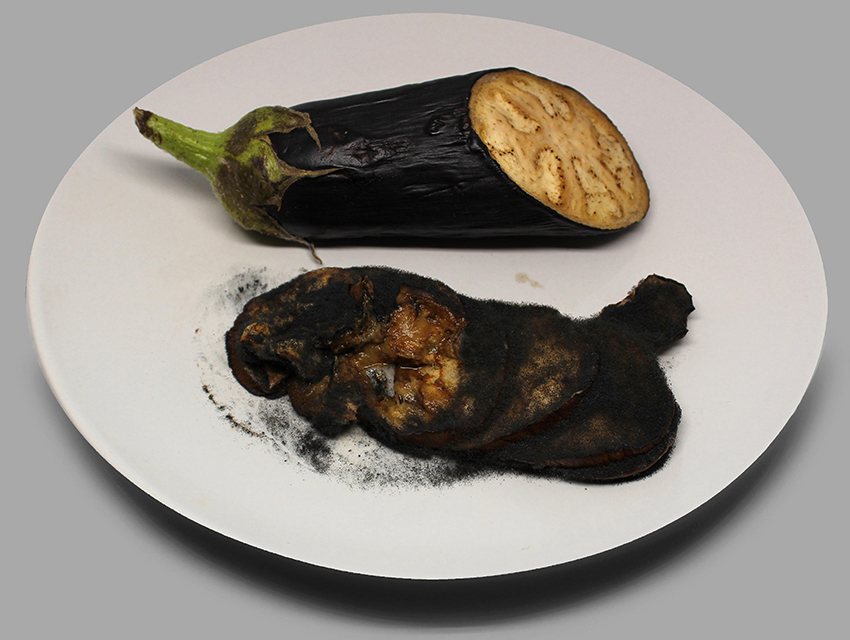

Supplement: Supplementary file 1 [file foods-10-01440-s001.zip › SupplementaryFiles/SuppBFoodImages/SubsetB/Unpalatable/tp0961.jpg]

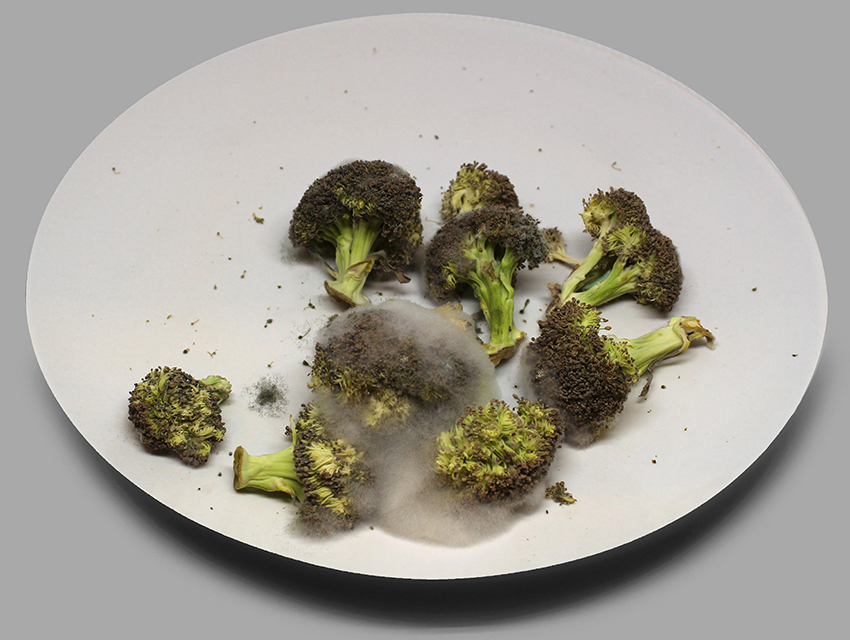

Supplement: Supplementary file 1 [file foods-10-01440-s001.zip › SupplementaryFiles/SuppBFoodImages/SubsetB/Unpalatable/tp0977.jpg]
